# Sex-dependent interferon signaling contributes to female-biased vulnerability in Alzheimer’s disease

**DOI:** 10.1186/s12974-026-03840-0

**Published:** 2026-05-04

**Authors:** Verónica López-López, Gerard Iniesta, Marcos Galán-Ganga, Alejandro Expósito-Coca, Violeta Durán-Laforet, Aysha M. Bhojwani-Cabrera, Carmen M. Navarrón, Marina Guillot-Fernández, Jose L. Venero, Jose V. Sánchez-Mut, Angel Barco, Albert Giralt, José P. López-Atalaya

**Affiliations:** 1 Instituto de Neurociencias (IN), Consejo Superior de Investigaciones Científicas, Universidad Miguel Hernández, Sant Joan d’Alacant, Spain; 2 Departament de Biomedicina, Facultat de Medicina, Institut de Neurociències, Universitat de Barcelona, Barcelona, Spain; 3 Institut d’Investigacions Biomèdiques August Pi I Sunyer (IDIBAPS), Barcelona, Spain; 4 Centro de Investigación Biomédica en Red Sobre Enfermedades Neurodegenerativas (CIBERNED), Madrid, Spain; 5 Instituto de Biomedicina de Sevilla, IBiS/Hospital Universitario Virgen del Rocío/CSIC/Universidad de Sevilla, Seville, Spain; 6 Departamento de Bioquímica y Biología Molecular, Facultad de Farmacia, Universidad de Sevilla, Seville, Spain; 7 Production and Validation Centre of Advanced Therapies (Creatio), Faculty of Medicine and Health Science, University of Barcelona, Barcelona, Spain

**Keywords:** Alzheimer’s disease, Sex differences, Neuroinflammation, Interferon, Microglia, cGAS-STING pathway

## Abstract

Alzheimer’s disease (AD) disproportionately affects women, yet the biological basis of this sex bias remains unclear. Here, we identify sex-dependent interferon signaling as a contributor to this disparity. Transcriptomic profiling of *postmortem* AD tissue and APP/PS1 mice revealed preferential enrichment of interferon-responsive gene programs in females. In APP/PS1 mice, heightened interferon responses were associated with increased neurodegenerative features, and single-cell transcriptomic analyses identified microglia as a major cellular compartment engaging interferon responses. To test causality, we manipulated interferon signaling in vivo. Acute systemic interferon activation promoted AD-like neuropathological alterations. Genetic amplification of interferon signaling in microglia exacerbated neuroinflammatory and neurodegenerative features in APP/PS1 mice, whereas pharmacological inhibition through cGAS-STING blockade suppressed interferon responses, reduced neuropathology, and preserved cognitive performance in female APP/PS1 mice. Together, these findings identify microglial interferon signaling as a modifiable contributor to AD-associated neuropathology and suggest a neuroimmune mechanism underlying the increased vulnerability of females to the disease.

**Supplementary Information:**

The online version contains supplementary material available at 10.1186/s12974-026-03840-0.

## Background

**Fig. 1:**
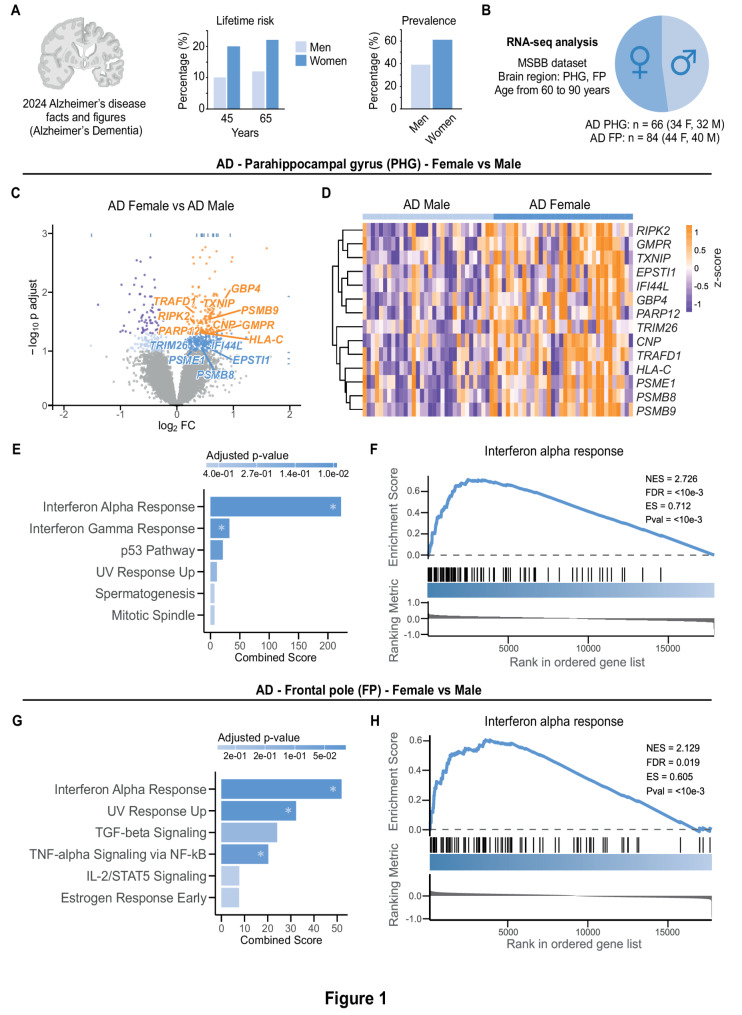
Enhanced interferon signaling associates with female-biased Alzheimer’s disease risk and prevalence. **A** Diagram illustrating the lifetime risk at 45 and 65 years of age, and prevalence of Alzheimer’s disease (AD) in women and men (Alzheimer’s Facts and Figs. 2024). **B** Schematic of the RNA sequencing (RNA-seq) cohorts from the Mount Sinai Brain Bank (MSBB), comprising parahippocampal gyrus (PHG) samples from 34 female and 32 male AD patients, and frontal pole (FP) samples from 44 female and 40 male AD patients (all aged 60–90 years). **C** Volcano plot showing differential gene expression analysis in the PHG of female (*n* = 34) versus male (*n* = 32) AD brains. Significantly upregulated genes (adj. *p* < 0.05) are shown in orange, and genes with 0.05 ≤ adj. *p* < 0.1 are shown in dark blue. Interferon-related genes are highlighted. **D** Heatmap showing the top interferon-related genes with sex-biased expression differences (adj. *p* < 0.1) in the PHG of female AD samples compared to male samples. **E** Functional enrichment analysis performed on significantly upregulated genes (adj. *p* < 0.05) in the PHG of female AD brains. **F** Gene set enrichment analysis (GSEA) performed on the ranked gene list derived from the differential expression analysis in (**C**), showing the MSigDB Hallmark “interferon alpha response” gene set. **G** Functional enrichment analysis using the top 200 upregulated genes ranked by adjusted *p*-value from differential expression analysis comparing female (*n* = 44) and male (*n* = 40) AD samples in the frontal pole (FP). **H** GSEA plot of the MSigDB Hallmark “interferon alpha response” gene set based on differential expression analysis in the FP

Alzheimer’s disease (AD) is a progressive and ultimately fatal neurodegenerative disorder and the most common cause of dementia worldwide [1–3]. With an aging global population, the number of individuals affected by AD is projected to exceed 100 million by 2050, placing an enormous burden on healthcare systems and caregivers [1, 3–5]. A consistently reported yet underexplored feature of AD is its disproportionate impact on females. Females comprise nearly two-thirds of individuals diagnosed with AD [1, 3, 5, 6], and lifetime risk estimates indicate that approximately 1 in 5 females, compared to 1 in 10 males, will develop the disease [1, 4] (Fig. 1A). Although increased longevity contributes to this imbalance, it does not fully account for the observed differences [7–10]. Moreover, clinical and neuroimaging studies have revealed that females with AD often exhibit faster cognitive decline, greater neuropathological burden, and more rapid progression from mild cognitive impairment to dementia than their male counterparts [7, 11–16].

Despite its clinical significance, the mechanistic basis for sex-related differences in AD remains poorly defined. The innate immune system—particularly neuroimmune signaling within glial networks—is increasingly recognized as a key contributor to AD pathogenesis. Genome-wide association studies (GWAS) have identified numerous AD susceptibility loci involved in immune regulation, including variants in microglial genes such as *TREM2*, and *CD33*, as well as *HLA* loci [17–20]. These discoveries have expanded the conceptual framework of AD beyond classical amyloid and TAU cascades, implicating immune dysregulation and glial biology as core features of disease pathogenesis [21–25]. In parallel, evidence show that immune responses are strongly shaped by biological sex. Females generally exhibit stronger baseline and stimulus-induced immune responses, both peripherally and in the central nervous system [26–30]. While this amplified responsiveness may enhance pathogen clearance, it is also linked to greater vulnerability to chronic inflammation and autoimmunity [26, 27, 29]. Whether such immunological differences translate into distinct molecular trajectories or pathological outcomes in AD remains an open question.

In this study, we sought to investigate the molecular mechanisms underlying sex-associated differences in AD vulnerability. We combined transcriptomic analysis of *postmortem* human brain tissue with functional studies in APP/PS1 mice to examine whether immune signaling pathways contribute to this disparity. Our analyses revealed a pronounced activation of interferon-responsive programs in female AD brains and pointed to microglia as a major cellular compartment engaging these responses. Using complementary pharmacological and genetic approaches in APP/PS1 mice, we further demonstrate that amplification of interferon signaling exacerbates neuroinflammatory and neurodegenerative features of the disease, whereas its inhibition mitigates neuropathological alterations and preserves cognitive function. Together, these findings identify interferon signaling as a key contributor to sex-associated differences in AD vulnerability and highlight this pathway as a potential therapeutic target.

## Results

### Sex-dependent interferon pathway activation in Alzheimer’s disease

To investigate sex-associated transcriptional differences in AD, we analyzed bulk RNA-sequencing data from the parahippocampal gyrus (PHG), a medial temporal lobe region that is among the earliest and most severely affected cortical areas in AD [31, 32], in individuals from The Mount Sinai Brain Bank (MSBB) study [33]. To minimize potential survival bias due to overrepresentation of women at the most advanced ages (> 90 years), analyses were restricted to individuals aged 60–90 years, yielding a cohort of 66 AD cases (34 females, 32 males) (Fig. 1B; Fig. S1, A and B; Table S1). To account for residual differences in age distribution between sexes (Fig. S1, C-J), age was included as a covariate in all differential expression analyses.

Differential gene expression analysis comparing female and male AD samples in the PHG revealed a prominent female-biased transcriptional signature enriched for interferon-related genes, including *GBP4, CNP, PSMB9, PSMB8, EPSTI1, IFI44L,* and *TRIM26* (Fig. 1, C and D; Table S1). Functional enrichment analysis identified “interferon alpha response” as the most significantly enriched “Hallmark” gene set [34] in females, followed by “interferon gamma response”, whereas “p53 pathway” showed borderline enrichment (adj. p = 0.05) (Fig. 1E). Gene Set Enrichment Analysis (GSEA) [35] further demonstrated coordinated upregulation of the “interferon alpha response” gene set in female AD samples (Fig. 1F). Notably, no significant differences were observed between females and males in amyloid burden or Braak stage within this cohort (Fig. S1C-F), indicating comparable neuropathological severity across sexes.

To determine whether this female-biased interferon signature extended beyond the medial temporal lobe, we analyzed bulk RNA-sequencing data from the frontal pole (FP) of individuals with AD from the same MSBB cohort (44 females and 40 males; Fig. 1B). Consistent with the PHG findings, enrichment analyses in the FP revealed significant upregulation of interferon-related pathways in females relative to males (Fig. 1G). GSEA further confirmed coordinated enrichment of the “interferon alpha response” gene set in female samples from the FP (Fig. 1H), indicating that the sex-dependent interferon signature extends beyond the medial temporal lobe.

To contextualize these findings in the broader molecular landscape of AD, we next compared AD and age-matched control samples without sex stratification (n = 108; 66 AD, 42 controls). In this pooled analysis, “epithelial mesenchymal transition” emerged as the top enriched pathway, followed by “interferon previ response” and “interferon alpha response”, alongside other established AD-associated pathways such as “cholesterol homeostasis” (Fig. S1K; Table S1). However, stratification by sex revealed distinct transcriptional architectures: in males (n = 55; 32 AD, 23 controls), “epithelial mesenchymal transition” was the dominant enriched pathway, whereas in females (n = 53; 34 AD, 19 controls), “interferon alpha response” became the leading enriched program (Fig. S1, L and M). These findings indicate that interferon-related transcriptional responses are preferentially engaged in females in the context of AD.

Finally, we examined whether this interferon signature was present under non-pathological conditions. Analysis of PHG and FP samples from age-matched control individuals (PHG: n = 42; 19 females, 23 males; FP: n = 50; 27 females and 23 males) revealed no significant enrichment of interferon-related pathways between sexes (Fig. S1N-P; Table S1). Thus, baseline sex differences in interferon signaling appear limited, whereas AD pathology is associated with a marked female-biased interferon gene expression signature.

Collectively, these data demonstrate a robust and regionally reproducible enrichment of type I interferon-associated transcriptional programs in female AD brains. This sex-dependent immune signature is observed in the absence of differences in classical neuropathological staging and suggests that interferon pathway engagement may contribute to female-biased vulnerability in AD.

### Sex-dependent neuropathological alterations in APP/PS1 mice

To determine whether the female-biased vulnerability observed in human AD is recapitulated in experimental models, we analyzed male and female APPswe/PSEN1dE9 (APP/PS1) mice. This model develops robust amyloid pathology and neuroinflammatory changes and is widely used to study AD mechanisms. Histological analyses were performed at 6 months of age, a stage when amyloid deposition is actively progressing in the hippocampus and cortex of APP/PS1 mice [36, 37].

**Fig. 2:**
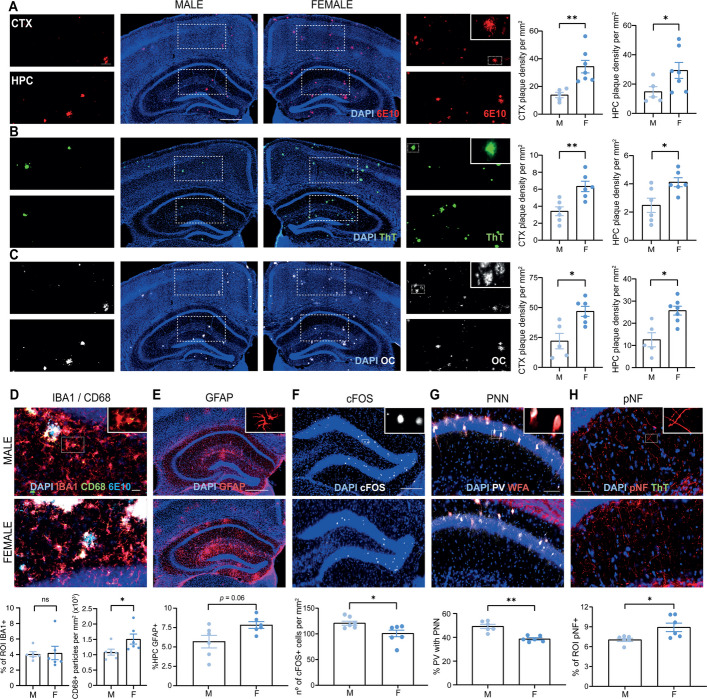
Female APP/PS1 mice exhibit increased amyloid burden and exacerbated histopathological alterations. **A**-**C** Representative images and quantification of amyloid-β (Aβ) plaque burden with 6E10 immunostaining (**A**), thioflavin T (ThT) staining (**B**), and OC immunostaining (**C**) in cortex (CTX) and hippocampus (HPC) of male and female APP/PS1 mice at 6 months of age (male, *n* = 5–6; female, *n* = 6–7). Nuclei were counterstained with DAPI. Scale bars: 500 μm; inset 100 μm. **D**-**H** Representative images and quantification of immunostaining in the hippocampus of male and female APP/PS1 mice at 6 months of age (*n* = 6 per group): IBA1, CD68, and 6E10 in CA1 stratum radiatum (**D**); GFAP in the hippocampus (**E**); cFOS in the dentate gyrus (**F**); PV and WFA labelling of perineuronal nets (PNNs) in CA1 (**G**); and pNF and ThT in the CA1 stratum radiatum (**H**). Nuclei were counterstained with DAPI. Scale bars: 20 μm (**D**); 500 μm (**E**); 200 μm (**F**); 100 μm (**G**); 50 μm (**H**). Graphs represent data distribution as dots and bars indicating mean ± SEM. Statistical significance was assessed using two-tailed Mann–Whitney U test (*p < 0.05; **p < 0.01)

Immunohistochemical staining using the anti-amyloid-β antibody 6E10 revealed a significantly higher plaque burden in both the cortex and hippocampus of female mice compared with males (Fig. 2A; Fig. S2). These findings were confirmed using thioflavin T staining, which detects fibrillar amyloid deposits (Fig. 2B), as well as OC immunostaining, which recognizes β-sheet-rich amyloid conformations (Fig. 2C). Across all three detection methods, females consistently exhibited higher amyloid plaque density. Notably, however, average plaque size was comparable between sexes (Fig. S3A-C), indicating that the observed sex difference primarily reflects increased plaque density rather than altered plaque morphology.

We next examined additional histopathological features associated with AD [38–42]. Immunostaining for microglial markers revealed that the area occupied by IBA1 staining was unchanged, whereas CD68 levels were increased, including higher density of CD68^+^ particles surrounding plaques, suggesting enhanced microglial phagocytic activity (Fig. 2D; Fig. S3D). Astrocytic reactivity also showed a trend toward higher GFAP immunoreactivity in females (Fig. 2E; Fig. S3E). These findings suggest that the female APP/PS1 brain exhibits an amplified neuroinflammatory response to amyloid pathology.

Consistent with these immune alterations, several markers of neuronal dysfunction were also more pronounced in females. cFOS staining in the dentate gyrus was reduced in female mice, suggesting altered neuronal activity (Fig. 2F). In parallel, the proportion of parvalbumin interneurons (PV^+^) surrounded by perineuronal nets (PNNs) in the CA1 region was significantly reduced in females, as visualized by *Wisteria floribunda* (WFA) agglutinin staining (Fig. 2G; Fig. S3F). Finally, phosphorylated neurofilament (pNF) staining revealed increased axonal damage in the hippocampus of female mice (Fig. 2H). Together, these observations indicate that female APP/PS1 mice exhibit more severe neuronal dysfunction and neuropathological alterations than males.

To establish the molecular landscape associated with amyloid pathology in this model, we performed bulk RNA sequencing of hippocampal tissue from APP/PS1 and wild-type mice. As expected, differential expression analysis identified strong upregulation of genes associated with the APP/PS1 transgene expression (*App* and *Prnp*) and microglial activation, including *Clec7a*, *Trem2*, *Cst7*, and *Itgax* (Fig. S3G; Table S2) [43–45]. Functional enrichment analysis further revealed activation of immune and metabolic pathways, including “cholesterol homeostasis”, “allograft rejection”, and “inflammatory response” (Fig. S3H), confirming that the APP/PS1 model displays the expected transcriptional signature of AD-associated neuroinflammation.

### Sex-dependent interferon signaling in APP/PS1 hippocampus

**Fig. 3:**
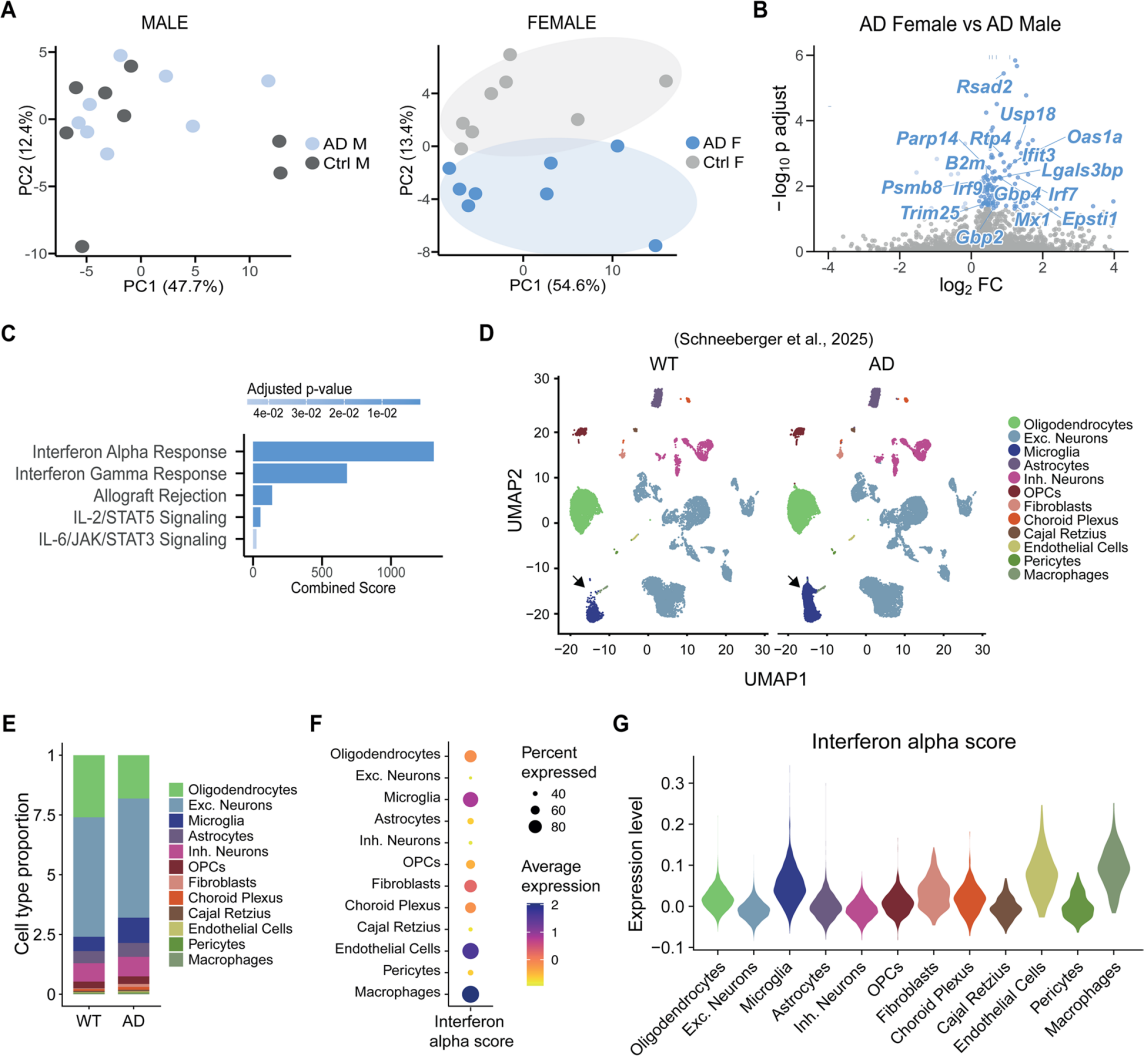
Interferon alpha signaling is increased in female APP/PS1 hippocampus and is enriched in microglia. **A** Principal component analysis (PCA) of bulk hippocampal RNA-seq data from 6-month-old male and female APP/PS1 (AD) and control (Ctrl) mice (AD M, *n* = 8; AD F, *n* = 8; Ctrl M, *n* = 8; Ctrl F, *n* = 8; M = male, F = female). **B** Volcano plot showing differential gene expression between female and male APP/PS1 hippocampus, highlighting upregulated interferon alpha-related genes (*n* = 8 per group). **C** Functional enrichment analysis of genes upregulated in female APP/PS1 hippocampus (adj. *p* < 0.05). **D**-**G** Analyses of hippocampal single-nucleus RNA sequencing (snRNA-seq) data from 8-month-old male APPPS1 mice (AD, *n* = 3; 25,213 high-quality nuclei) and wild-type littermate controls (WT, *n* = 2; 15,019 high-quality nuclei). **D** UMAP projection colored by cell-type annotation. **E** Stacked bar plots showing cell-type composition in AD and WT samples. **F** Dot plot showing “interferon alpha response” gene set module scores across cell types. Dot size indicates the percentage of nuclei expressing genes from the interferon alpha gene set, and color represents the average module score. **G** Violin plot showing the distribution of interferon alpha gene set module scores across cell types

Given the more severe neuropathological alterations observed in female APP/PS1 mice, we next asked whether amyloid pathology elicited sex-dependent transcriptional responses in the hippocampus. Principal component analysis (PCA) of hippocampal RNA-seq data revealed limited separation between APP/PS1 and wild-type samples in males, whereas APP/PS1 females diverged more clearly from their wild-type counterparts (Fig. 3A), suggesting a more pronounced transcriptional remodeling in females at this stage of disease.

To determine whether the interferon signature observed in female AD brains (Fig. 1) was recapitulated in the APP/PS1 model, we directly compared hippocampal transcriptomes from female and male APP/PS1 mice. Differential expression analysis revealed a prominent enrichment of interferon-stimulated genes in females, including *Gbp4*, *Psmb8*, *Epsti1*, *Irf7*, *Irf9*, *Ifit3*, *Oas1a*, and *Rtp4* (Fig. 3B; Table S2). Consistent with this observation, functional enrichment analysis identified “interferon alpha response” as the most significantly enriched Hallmark gene set in females, followed by “interferon gamma response” (Fig. 3C). GSEA further confirmed enrichment of the Hallmark “interferon alpha response” program in female APP/PS1 hippocampus (Fig. S3I).

Next, to determine which cell types contribute to this interferon signature, we examined publicly available hippocampal single-nucleus RNA-sequencing (snRNA-seq) data from 8-month-old APPPS1 and wild-type controls [46]. UMAP visualization confirmed the presence of major neural and glial cell populations (Fig. 3D). As reported in the original study, APPPS1 mice exhibited microglial expansion together with a reduction in oligodendrocytes (Fig. 3E) [46]. Interferon alpha gene signature was strongly enriched in microglia, macrophages, and endothelial cells (Fig. 3F,G; Fig. S3J,K). However, macrophage and endothelial populations were comparatively rare, whereas microglia constituted the most abundant interferon-responsive cell population in the hippocampus. These findings identify microglia as the principal cell type associated with interferon signaling in the APPPS1 hippocampus.

Together, these findings reveal a female-biased activation of interferon signaling in the APP/PS1 model and identify microglia as a major cell type associated with this response. This observation raised the possibility that interferon pathway activation may contribute to the development of AD-related neuropathology.

### Acute interferon signaling promotes Alzheimer’s disease-associated neuropathological alterations

**Fig. 4:**
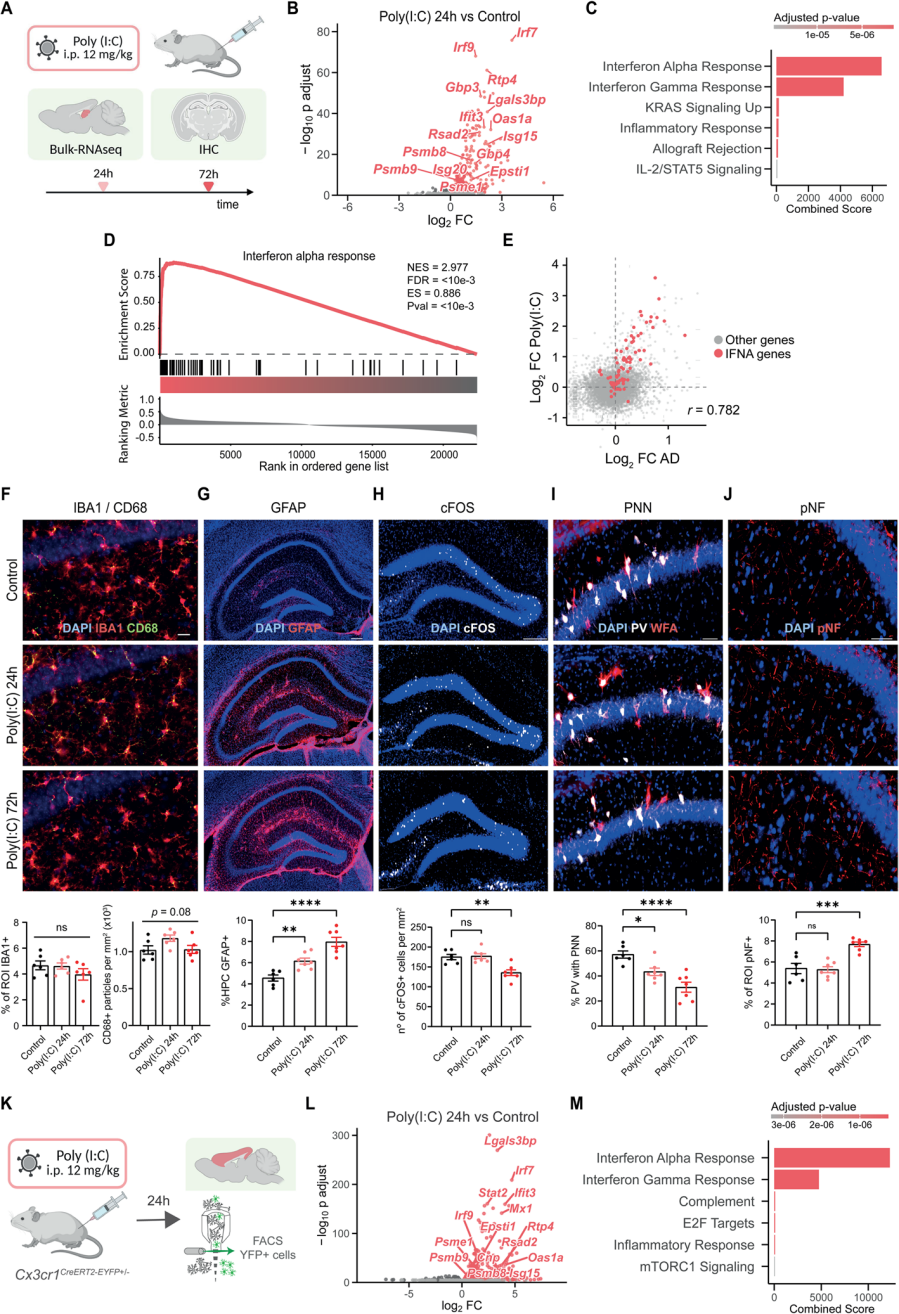
Acute interferon signaling elicits Alzheimer-like pathology. **A** Schematic of the experimental design showing intraperitoneal administration of poly(I:C) (12 mg/kg, i.p.) or saline vehicle to 3-month-old wild-type mice. Hippocampal bulk RNA sequencing (bulk RNA-seq) and immunohistochemistry (IHC) were performed at 24 and 72 h post-treatment. **B** Volcano plot of differential gene expression in the hippocampus 24 h post-poly(I:C) versus saline (control) (control: males, *n* = 5; females, *n* = 4; poly(I:C) 24 h: males, *n* = 4; females, *n* = 5). Red dots represent significantly upregulated genes (adj. *p* < 0.05), with interferon alpha-related genes highlighted. **C** Functional enrichment analysis of genes upregulated 24 h post-poly(I:C) (adj. p < 0.05). **D** GSEA plot of the MSigDB Hallmark “interferon alpha response” gene set based on differential expression analysis 24 h post-poly(I:C). **E** Scatter plot comparing log2 fold changes in the hippocampus of mice treated with poly(I:C) for 24 h and APP/PS1 mice (AD). Red dots represent interferon alpha gene set genes (IFNA genes; *n* = 89 genes); grey dots represent all other genes. Pearson correlation for interferon alpha genes: *r* = 0.782. **F**-**J** Representative images and quantification of immunostaining in saline-treated control mice and mice treated with poly(I:C) for 24 h or 72 h: IBA1 and CD68 in the stratum radiatum (**F**); GFAP in the hippocampus (**G**); cFOS in the dentate gyrus (**H**); PV and WFA labelling of perineuronal nets (PNNs) in CA1 (**I**); pNF in the stratum radiatum (**J**). Nuclei were counterstained with DAPI. Scale bars: 20 μm (**F**); 500 μm (**G**); 200 μm (H); 100 μm (**I**); 50 μm (**J**). Sample size for IHC in (**F**): control, *n* = 6 (4 males, 2 females); poly(I:C) 24 h, *n* = 6 (3 males, 3 females); poly(I:C) 72 h, *n* = 6 (3 males, 3 females). Sample size for IHC in (**G**-**J**): control, *n* = 6 (4 males, 2 females); poly(I:C) 24 h, *n* = 7 (4 males, 3 females); poly(I:C) 72 h, *n* = 7 (4 males, 3 females). Control animals include mice analyzed at both 24 h and 72 h post-injection. Graphs represent data distribution as dots and bars indicating mean ± SEM. Statistical significance was assessed using one-way ANOVA with Bonferroni post hoc test (ns, not significant; *p < 0.05; **p < 0.01; ***p < 0.001; ****p < 0.0001). **K** Schematic of microglial isolation: Cx3cr1.^+^ microglial cells were acutely isolated by fluorescence-activated cell sorting (FACS) from adult mouse brains 24 h after poly(I:C) administration (12 mg/kg, i.p.). **L** Volcano plot of differential gene expression in FACS-isolated microglia following systemic poly(I:C) (poly(I:C), *n* = 3; control, *n* = 4). **M** Functional enrichment analysis of significantly upregulated genes (adj. *p* < 0.05) in microglia after poly(I:C)

Given the association between interferon signaling and neuropathological alterations observed in both human AD samples and APP/PS1 mice, we next examined whether acute activation of interferon responses is sufficient to promote similar pathological features. To test this hypothesis, adult wild-type C57BL/6 mice were administered a single intraperitoneal injection of polyinosinic:polycytidylic acid [poly(I:C); 12 mg/kg], a synthetic double-stranded RNA analog that activates pattern-recognition receptors such as TLR3 and MDA5 and induces a robust type I interferon response. Brain tissue was then analyzed 24 or 72 h post-injection (Fig. 4A). Both male and female mice were included in the experiments, and no major sex-dependent differences were observed; therefore, data from both sexes were analyzed together.

To characterize the transcriptional response induced by systemic interferon activation, we performed bulk RNA sequencing of hippocampal tissue 24 h after poly(I:C) administration (Fig. 4A; Fig. S4A). Differential expression analysis revealed strong upregulation of interferon-stimulated genes, including *Gbp4*, *Psmb9*, *Psmb8*, *Epsti1*, *Irf7*, *Irf9*, *Ifit3*, *Oas1a*, and *Rtp4* (Fig. 4B; Table S3). Functional enrichment analysis identified “interferon alpha response” as the most significantly enriched Hallmark gene set, followed by “interferon gamma response” (Fig. 4C). Gene set enrichment analysis (GSEA) further confirmed robust activation of the “interferon alpha response” program (Fig. 4D).

Notably, the transcriptional response induced by poly(I:C) closely resembled the interferon signature observed in the APP/PS1 model. Expression changes in interferon-responsive genes showed a strong positive correlation between poly(I:C)-treated hippocampus and AD samples (Fig. 4E), indicating that systemic interferon activation recapitulates key aspects of the AD-associated interferon program.

We next examined hippocampal neuropathological features following systemic interferon activation. Microglial responses were first assessed using IBA1 and CD68 immunostaining. While microglial density remained unchanged following poly(I:C) challenge, CD68^+^ particles transiently increased, suggesting enhanced microglial phagolysosomal activity. Poly(I:C) administration elicited marked astrocytic reactivity, as evidenced by increased GFAP immunoreactivity (Fig. 4G; Fig. S4C). cFOS staining in the dentate gyrus was reduced, suggesting altered neuronal activity (Fig. 4H). Inhibitory network integrity was also affected, with a reduction in the proportion of parvalbumin-positive interneurons surrounded by perineuronal nets (Fig. 4I; Fig. S4D). Finally, axonal damage increased, as indicated by elevated pNF staining (Fig. 4J). Together, these results revealed a temporally staged response to poly(I:C) challenge, with early astrocytic and extracellular matrix alterations detectable at 24 h, followed by changes in neuronal activity and axonal damage markers at 72 h. Overall, these observations indicate that acute activation of interferon signaling is sufficient to promote neuropathological alterations in the hippocampus that resemble key features observed in the APP/PS1 model.

To determine whether microglia directly engage this interferon program, we performed bulk RNA-sequencing of acutely isolated microglia from adult Cx3cr1::CreERT2-EYFP mice 24 h after poly(I:C) administration. Microglia were isolated by fluorescence-activated cell sorting based on YFP expression (Fig. 4K; Fig. S4E). Principal component analysis revealed clear separation between saline- and poly(I:C)-treated microglial transcriptomes (Fig. S4F), indicating a robust transcriptional response. Differential expression analysis revealed strong induction of interferon-stimulated genes in microglia, including *Gbp4*, *Cnp*, *Psmb9*, *Psmb8*, *Epsti1*, *Irf7*, *Irf9*, *Ifit3*, *Oas1a*, and *Rtp4* (Fig. 4L; Table S4). Consistent with these findings, pathway enrichment analysis identified “interferon alpha response” as the dominant transcriptional program (Fig. 4M), and gene set enrichment analysis further confirmed enrichment of the “interferon alpha response” program in poly(I:C)-treated microglia (Fig. S4G). These findings demonstrate that systemic poly(I:C) challenge induces a robust interferon response in microglia, indicating that this cell type actively engages interferon signaling in the brain following innate immune activation.

### Microglial amplification of interferon signaling exacerbates Alzheimer’s-associated neuropathology

**Fig. 5:**
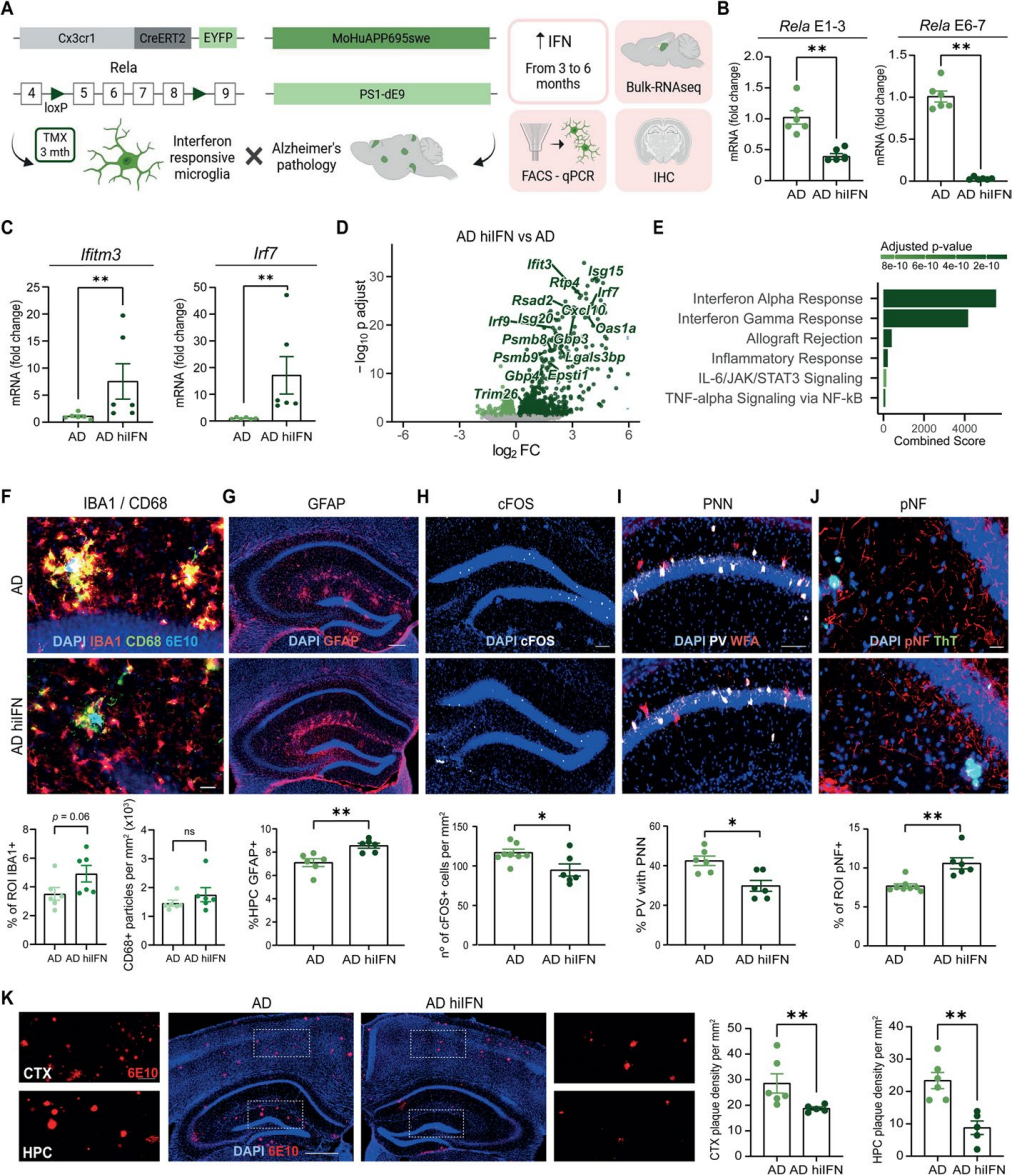
Genetic enhancement of interferon signaling exacerbates Alzheimer’s disease-like pathology. **A** Schematic of the experimental design. APP/PS1 (AD) mice were crossed with a microglia-specific *Rela* conditional knockout line (AD hiIFN). Tamoxifen was administered at 3 months of age, and analyses were performed at 6 months, including quantitative PCR (qPCR) of FACS-isolated microglia, hippocampal bulk RNA-sequencing (RNA-seq), and immunohistochemistry (IHC). **B** qPCR analysis of *Rela* expression in microglia from AD and AD hiIFN mice (*n* = 6 per group; 3 males and 3 females). Primers were designed to amplify *Rela* transcripts spanning exons 1–3 or exons 6–7. **C** qPCR analysis of *Ifitm3* and *Irf7* expression in acutely isolated microglia from AD and AD hiIFN mice (*n* = 6 per group; 3 males and 3 females). **D** Volcano plot of differentially expressed genes identified by bulk RNA-seq of hippocampal tissue comparing AD and AD hiIFN mice (males, *n* = 4; females, *n* = 4 per group). Dark green and light green dots represent significantly upregulated and downregulated genes, respectively (adj. *p* < 0.05). Interferon alpha-related genes among the upregulated set are highlighted. **E** Functional enrichment analysis of genes upregulated in AD hiIFN versus AD hippocampus (adj. *p* < 0.05). **F**-**J** Representative images and quantification of immunostaining in the hippocampus of AD and AD hiIFN mice: IBA1, CD68, and 6E10 in the stratum radiatum (**F**); GFAP in the hippocampus (**G**); cFOS in the dentate gyrus (**H**); PV and WFA labelling of perineuronal nets (PNNs) in CA1 (**I**); pNF in the stratum radiatum (**J**). Nuclei were counterstained with DAPI. Scale bars: 20 μm (**F**); 500 μm (**G**); 200 μm (**H**); 100 μm (**I**); 20 μm (**J**). Sample size for IHC in (**F**, **G**): AD, *n* = 6 (3 males, 3 females); AD hiIFN, *n* = 6 (3 males, 3 females). Sample size for IHC in (**H**-**J**): AD, *n* = 8 (4 males, 4 females); AD hiIFN, *n* = 6 (3 males, 3 females). **K** Representative images and quantification of 6E10 immunostaining for amyloid-β (Aβ) plaques in the cortex (CTX) and hippocampus (HPC) of AD and AD hiIFN mice. Nuclei were counterstained with DAPI. Scale bars: 500 μm; inset 100 μm. Sample size for IHC in (**K**): AD, *n* = 6 (3 males, 3 females); AD hiIFN, *n* = 5 (2 males, 3 females). Graphs represent data distribution as dots and bars indicating mean ± SEM. Statistical significance was assessed using two-tailed Mann–Whitney U test (ns, not significant; *p < 0.05; **p < 0.01)

Given the strong interferon response observed in microglia following innate immune activation, we next asked whether microglial interferon signaling contributes to AD-associated neuropathology. Perturbations in NF-κB signaling have been linked to the induction of interferon-stimulated genes in myeloid cells, and dominant-negative mutations in *RELA* in humans lead to a type I interferonopathy characterized by elevated expression of interferon-response genes [47, 48]. Consistent with this framework, we recently showed that *Rela* deletion in microglia drives transcriptional reprogramming toward an interferon-responsive state [49]. To assess the impact of sustained microglia-driven interferon activation on AD progression, we generated a gain-of-function model by crossing APP/PS1 mice with a tamoxifen-inducible, microglia-specific *Rela* knockout line (Cx3cr1::CreERT2-EYFP; Rela^fl/fl^), hereafter referred to as AD hiIFN (Fig. 5A). Mice were sacrificed at 6 months of age, allowing us to evaluate the consequences of chronic microglia-driven interferon activation during a critical stage of Alzheimer’s pathology progression. Mice of both sexes were included in the analysis, and no consistent sex-dependent differences were observed; therefore, data from males and females were analyzed together.

Efficient *Rela* deletion in FACS-isolated microglia was confirmed by qPCR, showing reduced expression across *Rela* exons (Fig. 5B). This genetic manipulation resulted in increased expression of interferon-associated transcripts in microglia, including *Ifitm3* and *Irf7* (Fig. 5C). At the tissue level, bulk hippocampal RNA-seq revealed robust upregulation of the interferon-stimulated gene program in AD hiIFN mice compared with AD controls (Fig. 5D; Fig. S5A; Table S5). Canonical interferon-stimulated transcripts upregulated in AD hiIFN included *Gbp4*, *Psmb9*, *Psmb8*, *Epsti1*, *Irf7*, *Irf9*, *Ifit3*, *Oas1a*, *Rtp4*, *Rsad2*, and *Isg15*. Pathway-level analyses identified “interferon alpha response” as the most significantly enriched Hallmark gene set, with additional enrichment of “interferon gamma response” (Fig. 5E; Fig. S5B and C), consistent with a strong interferon-responsive state.

We next assessed AD-relevant neuropathological features. AD hiIFN mice displayed exacerbated glial activation, with increased GFAP immunoreactivity in the hippocampus and a similar trend in the cortex, together with a modest increase in microglial activation (Fig. 5F and G; Fig. S5D and E). Consistent with heightened neuroinflammatory responses, AD hiIFN mice exhibited several markers of neuronal dysfunction. In the dentate gyrus, cFOS staining was further reduced compared with AD controls, suggesting impaired neuronal activity (Fig. 5H). Structural alterations in inhibitory circuits were also evident, with a reduced proportion of PV^+^ interneurons ensheathed by perineuronal nets in CA1 (Fig. 5I; Fig. S5F). Markers of axonal injury were likewise increased, as indicated by elevated pNF staining in the hippocampus (Fig. 5J), supporting enhanced neurodegenerative changes in AD hiIFN mice.

Interestingly, despite these exacerbated neuroinflammatory and neurodegenerative alterations, amyloid plaque burden was reduced in AD hiIFN mice. Quantification of amyloid plaques using 6E10 immunostaining (Fig. 5K), Thioflavin T labeling (Fig. S5H), and OC immunoreactivity (Fig. S5I) revealed lower plaque density in both cortex and hippocampus, while plaque size distribution remained largely unchanged (Fig. S5G, J, and K). Together, these results indicate that microglia-driven amplification of an interferon-responsive state contributes to multiple AD-associated neuropathological features while partially uncoupling neurodegenerative alterations from plaque burden.

### Modulation of interferon signaling via STING antagonism ameliorates Alzheimer’s-associated histopathology

Previous studies have implicated interferon signaling in AD pathology [50–53], and the microglial cGAS-STING pathway has recently emerged as a potential driver of interferon responses in AD [54–56]. We therefore asked whether pharmacological inhibition of this pathway could exert neuroprotective effects in APP/PS1 female mice.

To inhibit cGAS-STING signaling, we used the selective STING antagonist C-176 [57]. We first confirmed the efficacy of C-176 in vitro, showing that the compound effectively suppressed the transcriptional response induced by poly(I:C) stimulation in BV2 microglial cultures (Fig. S6A).

**Fig. 6:**
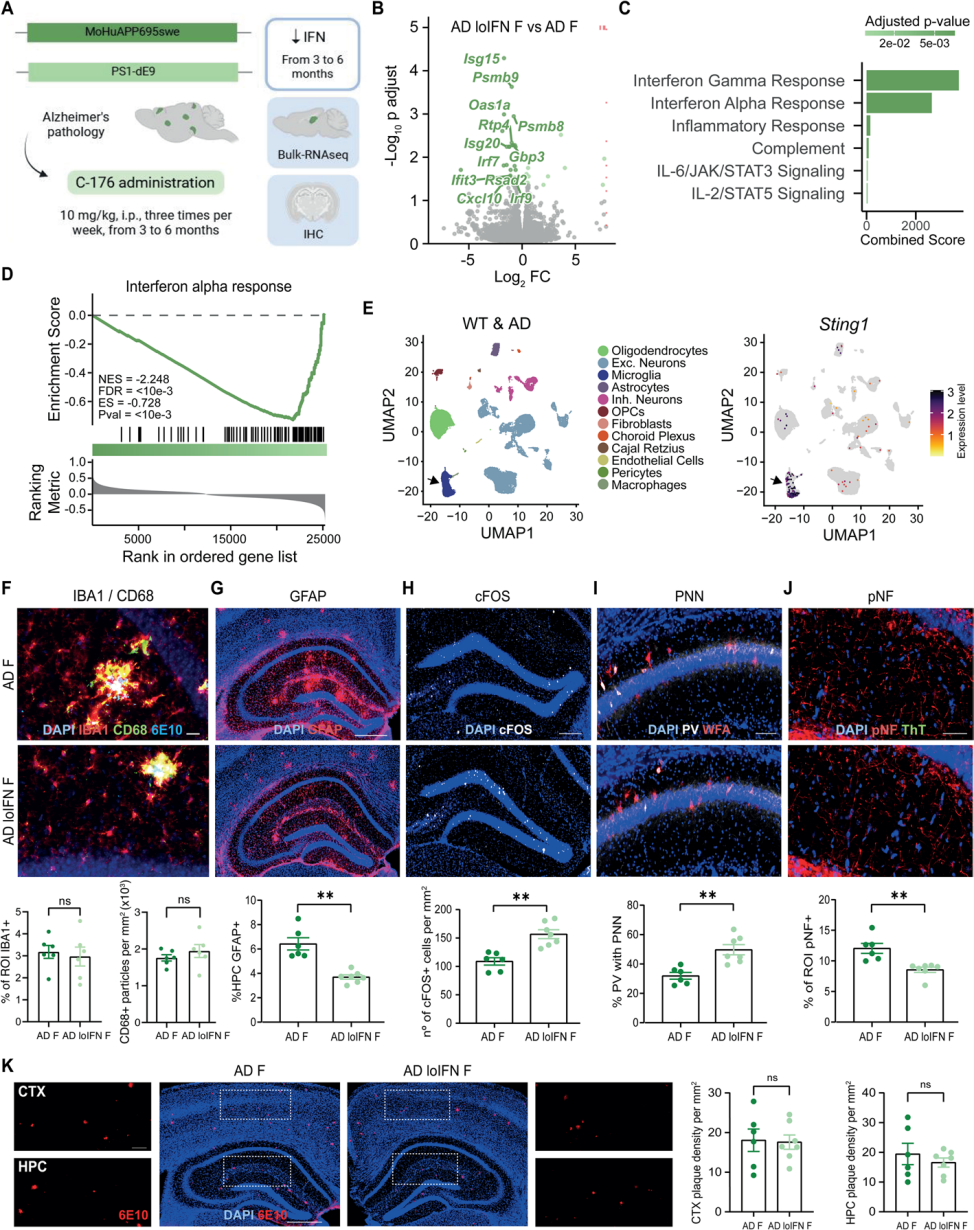
STING inhibition alleviates Alzheimer’s disease-like pathology in female APP/PS1 mice. **A** Schematic of experimental design for hippocampal bulk RNA-seq (RNA-seq) and immunohistochemistry (IHC) analyses in APP/PS1 mice treated with STING inhibitor C-176 (AD loIFN) or vehicle (AD). Three-month-old APP/PS1 mice received intraperitoneal administrations of C-176 (10 mg/kg) or vehicle three times per week until six months of age. **B** Volcano plot of differentially expressed genes identified by bulk RNA-seq of hippocampal tissue comparing AD loIFN versus AD female mice (AD F, *n* = 8; AD loIFN F, *n* = 4). Dark green and light green dots represent significantly downregulated and upregulated genes, respectively (adj. *p* < 0.05). Interferon alpha-related genes among the downregulated set are highlighted. **C** Functional enrichment analysis of genes downregulated in AD loIFN versus AD female hippocampus (adj. *p* < 0.05). **D** GSEA plot of the MSigDB Hallmark “interferon alpha response” gene set based on differential expression in AD loIFN versus AD female mice. **E** UMAP projection colored by cell-type annotation of hippocampal single-nucleus RNA sequencing (snRNA-seq) data from 8-month-old APPPS1 mice (AD, *n* = 3; 25,213 high-quality nuclei) and wild-type littermate controls (WT, *n* = 2; 15,019 high-quality nuclei) (left; related to Fig. 3D). Feature plot showing normalized *Sting1* expression (right). **F**-**J** Representative images and quantification of immunostaining in the hippocampus of AD and AD loIFN female mice: IBA1, CD68, and 6E10 in the stratum radiatum (**F**); GFAP in the hippocampus (**G**); cFOS in the dentate gyrus (**H**); PV and WFA labelling of perineuronal nets (PNNs) in CA1 (**I**); pNF in the stratum radiatum (**J**). Nuclei were counterstained with DAPI. Scale bars: 20 μm (**F**); 500 μm (**G**); 200 μm (**H**); 100 μm (**I**); 20 μm (**J**). **K** Representative images and quantification of amyloid-β (Aβ) plaque burden assessed by 6E10 immunostaining in the cortex (CTX) and hippocampus (HPC) of AD and AD loIFN female mice. Nuclei were counterstained with DAPI. Scale bars: 500 μm; inset 100 μm. Sample size for IHC in (**F**): *n* = 6 per group. Sample size for IHC in (**G**-**K**): AD F, *n* = 6; AD loIFN F, n = 7. Graphs represent data distribution as dots and bars indicating mean ± SEM. Statistical significance was assessed using two-tailed Mann–Whitney U test (ns, not significant; **p < 0.01)

APP/PS1 mice were then treated with C-176 from 3 to 6 months of age, a period corresponding to early plaque development (Fig. 6A). These animals are hereafter termed AD loIFN. Gene expression profiling of hippocampal tissue from AD loIFN mice revealed a marked sex-dependent transcriptional response (Fig. 6; Fig. S6 and Fig. S7). APP/PS1 females showed robust suppression of interferon-responsive genes, including *Psmb9*, *Psmb8*, *Irf7*, *Irf9*, *Ifit3*, *Oas1a*, *Rtp4*, *Rsad2*, and *Isg15* (Fig. 6B and Table S6). Pathway enrichment and gene set enrichment analyses confirmed suppression of interferon-related programs following C-176 treatment (Fig. 6C and D). In contrast, male APP/PS1 mice showed minimal transcriptional changes following treatment (Fig. S6B; Fig. S7A and B). Analysis of cell-type-resolved expression profiles form a published snRNA-seq dataset indicated that *Sting* (*Tmem173*) is highly enriched in microglia relative to other brain cell types (Fig. 6E), consistent with the possibility that the transcriptional effects of C-176 involve modulation of microglial signaling.

We next examined the impact of STING inhibition on AD-relevant neuropathological features. APP/PS1 females treated with C-176 showed attenuated gliosis, as revealed by significant reduction in the GFAP immunoreactivity in the hippocampus and a similar trend observed in cortex, whereas microglial markers IBA1 and CD68 were unaltered (Fig. 6F and G; Fig. S6C and D). In parallel, cFOS expression in the dentate gyrus was partially restored and an increased proportion of PNN-ensheathed parvalbumin-positive interneurons was detected in CA1 (Fig. 6H and I; Fig. S6E). Likewise, axonal degeneration was reduced, as evidenced by a decrease in pNF staining in the hippocampus (Fig. 6J). In contrast, C-176 treatment produced more modest effects in male APP/PS1 mice, with significant changes observed only in selected parameters such as PNN and pNF, while other measures showed no significant differences (Fig. S7C-G).

Amyloid plaque burden remained largely unchanged following C-176 treatment. Quantification using 6E10 immunostaining (Fig. 6K; Fig. S6F), Thioflavin T labeling (Fig. S6G and I), and OC immunoreactivity (Fig. S6H and J) revealed no significant differences in plaque density or size in either cortex or hippocampus. Similar results were observed in male APP/PS1 mice (Fig. S7H-M).

Together, these findings indicate that STING inhibition attenuates interferon-associated neuroinflammatory and neurodegenerative features in APP/PS1 female mice while leaving amyloid plaque burden unaffected.

### STING inhibition protects cognitive function in female APP/PS1 mice

**Fig. 7:**
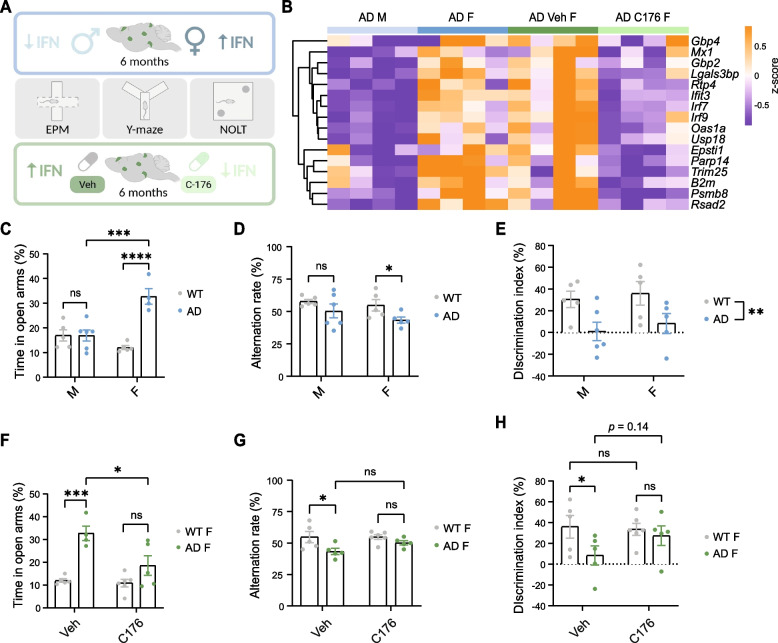
STING inhibition preserves cognitive performance in female APP/PS1 mice. **A** Schematic of the experimental design for cognitive testing in male (M) and female (F) APP/PS1 and wild-type littermate (WT) mice treated with the STING inhibitor C-176 (10 mg/kg, i.p.; AD loIFN) or vehicle (AD) from 3 to 6 months of age. **B** Heatmap showing the top interferon-alpha-related genes upregulated (adj. *p* < 0.05) in the hippocampus of female versus male AD mice (see Fig. 3B), together with hippocampal samples from female AD mice treated with C-176 or vehicle (*n* = 4 per group). **C**-**E** Behavioral assessment of male and female AD mice and wild-type littermates treated with vehicle (AD: males, *n* = 6; females, *n* = 4–5; WT: males, *n* = 5; females, *n* = 5). **C** Time spent in the open arms of the elevated plus maze (EPM). **D** Alternation rate in the Y-maze. **E** Discrimination index in the novel object location test (NOLT). **F**–**H** Behavioral assessment of female AD mice and WT littermates treated with C-176 or vehicle (AD: C-176, *n* = 5; vehicle, *n* = 4–5; WT: C-176, *n* = 6; vehicle, *n* = 5). **F** Time spent in the open arms of the elevated plus maze (EPM). **G** Alternation rate in the Y-maze. **H** Discrimination index in the novel object location test (NOLT). Vehicle-treated animals shown in panels **F**–**H** are the same animals used for the WT vs AD baseline comparisons shown in panels **C**-**E**. Graphs represent data distribution as dots and bars indicating mean ± SEM. Statistical significance was assessed using two-way ANOVA with Tukey’s HSD (**C**, **D**, **F**, **G**) or Fisher’s LSD post hoc test (**E**, **H**) (ns, not significant; *p < 0.05; **p < 0.01; ***p < 0.001; ****p < 0.0001)

Given that pharmacological inhibition of the cGAS-STING pathway suppresses interferon signaling and attenuates neuropathological alterations in APP/PS1 female mice, we next examined whether these changes translate into improved cognitive function (Fig. 7A and B).

Behavioral testing revealed domain-specific impairments in APP/PS1 mice with a clear sex bias. In the elevated plus maze (EPM), APP/PS1 males performed similarly to wild-type controls, whereas APP/PS1 females spent significantly more time in the open arms compared with wild-type females, indicating altered exploratory behavior in this task (Fig. 7C). Similarly, spontaneous alternation in the Y-maze was reduced in APP/PS1 females but not in males, reflecting spatial working memory deficits selectively affecting females (Fig. 7D). In the novel object location test (NOLT), both male and female APP/PS1 mice showed reduced discrimination index values compared with wild-type controls, indicative of impaired spatial memory performance (Fig. 7E; Fig. S8A). No significant differences were detected between genotypes or sexes in additional behavioral tasks, including the open field test, T-maze, contextual fear conditioning, and cued fear conditioning (Fig. S8B-E). This behavioral profile is consistent with previous reports that cognitive deficits in APP/PS1 mice begin to emerge around this age and remain task-dependent during early stages of pathology [36, 58].

We next assessed whether pharmacological suppression of interferon signaling could mitigate cognitive impairments in APP/PS1 females. Consistent with the molecular and histopathological improvements observed following STING inhibition, C-176 treatment improved behavioral performance across multiple tasks (Fig. 7F-H; Fig. S8F-R). In the EPM, treated APP/PS1 females spent less time in the open arms compared with vehicle-treated transgenic mice, restoring exploratory behavior to levels comparable with wild-type controls (Fig. 7F). In the Y-maze, C-176–treated females exhibited improved spontaneous alternation, indicating enhanced spatial working memory performance (Fig. 7G). Likewise, in the NOLT, treated mice showed increased discrimination index values relative to untreated APP/PS1 females, reflecting improved spatial memory performance (Fig. 7H; Fig. S8F).

Together, these findings indicate that pharmacological inhibition of the cGAS-STING pathway improves cognitive performance in female APP/PS1 mice, linking interferon signaling to behavioral impairments associated with AD.

## Discussion

Alzheimer’s disease (AD) disproportionately affects women, both in prevalence and progression rate [1, 3, 59, 60]. While demographic factors such as increased lifespan contribute to this disparity, growing evidence suggests that biological sex fundamentally shapes the neurobiological response to AD pathology [10, 61]. In this study, we identify interferon signaling as a sex-biased component of AD pathobiology. We show that interferon-responsive pathways are preferentially enriched in the brains of female AD patients and that this immune signature is recapitulated in the APP/PS1 mouse model. Through experimental manipulation of interferon signaling in vivo, we further demonstrate that amplification of this pathway exacerbates neuroinflammatory and neurodegenerative features of AD, whereas pharmacological suppression mitigates neuropathological alterations and preserves cognitive function. Our findings further point to microglia as a key cellular compartment engaging interferon responses and indicate that interferon-driven tissue alterations can occur largely independently of amyloid plaque burden.

Our transcriptomic analyses of the parahippocampal gyrus from AD patients revealed preferential enrichment of interferon-responsive gene programs in females. Both interferon alpha and interferon gamma pathways were represented among the most significantly upregulated programs. These signaling pathways, which are central mediators of antiviral immunity and innate immune activation, have increasingly been implicated in neurodegenerative disease [50–52, 62–67]. Our findings extend previous reports of sex differences in peripheral immune responses [28, 30, 68–70] to the human brain and suggest that female-biased amplification of innate immune signaling may contribute to increased susceptibility to AD [71–76].

Using the APP/PS1 model of amyloidosis, we observed a parallel sex bias in disease-associated immune activation. Female transgenic mice exhibited more pronounced neuropathological alterations than males, including increased gliosis, neuronal dysfunction, and axonal damage. These histopathological changes were accompanied by stronger induction of interferon-stimulated genes in the hippocampus of female mice. Notably, single-cell transcriptomic profiling further identified microglia as a major cellular compartment engaging interferon responses. Although sex differences in AD models have been described previously [44, 77–82], our findings provide mechanistic insight by linking female-biased interferon signaling with downstream neuropathological consequences.

To directly assess the functional impact of interferon activation in the brain, we administered the double-stranded RNA analog poly(I:C) systemically to wild-type mice. Transcriptomic analyses revealed that microglia mount a robust interferon response following poly(I:C) stimulation, consistent with their role as key innate immune sensors in the brain. This manipulation recapitulated several pathological features observed in AD models, including reduced neuronal activity, degradation of perineuronal nets, astrogliosis, and axonal damage [38, 82]. These findings are consistent with previous studies showing that interferon signaling can impair neuronal plasticity and cognitive function. For example, interferon responses have been linked to synaptic loss, suppression of hippocampal neurogenesis, and disruption of long-term potentiation in aging and neurodegenerative contexts [83–87]. Our observation that both cFOS-positive neurons and perineuronal nets decline following interferon activation further supports the idea that sustained interferon signaling can directly disrupt neuronal circuit integrity [51, 52, 83, 88–90].

To investigate the consequences of sustained interferon activation in a disease context, we employed a genetic gain-of-function model in which deletion of *Rela* in microglia promotes an interferon-responsive transcriptional state [49]. When combined with APP/PS1 pathology, this manipulation led to exacerbated neuroinflammation, axonal injury, and neuronal dysfunction. Notably, amyloid plaque burden was reduced in these animals despite the presence of more severe neuropathological alterations. These findings suggest that interferon signaling can drive tissue damage through mechanisms that are not strictly dependent on plaque accumulation [51, 52, 67]. This observation aligns with a growing body of evidence indicating that maladaptive immune responses, rather than protein aggregation alone, represent critical determinants of disease progression [22–24]. A similar phenotype has been reported in microglia-specific *C9orf72* knockout mice, which also show a strong interferon signature accompanied by synaptic loss and reduced amyloid deposition [91].

Mechanistically, growing evidence identifies the microglial cGAS-STING pathway as an important upstream driver of interferon signaling in Alzheimer’s disease. This innate immune pathway senses cytosolic DNA released during cellular stress, including mitochondrial DNA leakage occurring near amyloid plaques [54, 92–94]. Activation of cGAS-STING has been reported in both human AD brain tissue and experimental models, where it promotes interferon responses and neuroinflammation [53, 55, 56, 88, 95]. Consistent with this framework, our single-cell transcriptomic analyses indicate that *Sting1* expression is enriched in microglia relative to other brain cell types, suggesting that this cellular compartment may represent a major site of cGAS–STING activation in AD. We therefore targeted STING pharmacologically using the selective antagonist C-176. In APP/PS1 mice, STING inhibition suppressed interferon-stimulated gene expression, reduced gliosis, preserved neuronal activity and axonal integrity, and improved cognitive performance. Notably, these beneficial effects were most pronounced in APP/PS1 female mice, which displayed higher baseline interferon activity. Importantly, these improvements occurred despite minimal changes in amyloid plaque burden, reinforcing the concept that interferon-driven neurotoxicity can occur independently of plaque accumulation.

This dissociation between plaque load and clinical outcome parallels observations in so-called “resilient” individuals—older adults who harbor substantial amyloid pathology but remain cognitively intact [96, 97]. *Postmortem* studies of such individuals frequently reveal reduced glial activation and lower inflammatory signatures compared with patients who develop dementia [98, 99]. Our findings suggest that modulation of interferon signaling may influence whether amyloid pathology progresses toward overt neurodegeneration.

Although these results nominate interferon signaling as a potential therapeutic target, several considerations are relevant for clinical translation. The STING antagonist C-176 used in this study selectively targets the murine STING isoform [57]. Other compounds with activity against human STING, such as H-151, have been developed and may provide a more suitable basis for therapeutic exploration [57]. Importantly, preclinical studies indicate that several STING inhibitors can penetrate the central nervous system and modulate neuroinflammatory responses in vivo [88, 100, 101]. At the same time, interferon signaling plays critical roles in antiviral defense and tumor surveillance, raising potential concerns about systemic immunosuppression [90, 102–104]. Nevertheless, multiple agents targeting interferon pathways—including JAK inhibitors and antibodies against IFNAR—are already in clinical use for autoimmune diseases, suggesting that modulation of this axis may be feasible with appropriate patient selection and monitoring [105–107].

Finally, our findings highlight the importance of considering sex as a biological variable in both disease mechanisms and therapeutic strategies. Historically, females have been underrepresented in neuroscience research, and many preclinical studies have relied predominantly on male animals [9, 108–110]. In our study, the inclusion of both sexes revealed pronounced differences in interferon activation, neuropathological progression, and therapeutic response. The observation that STING inhibition provided stronger benefits in females underscores the possibility that sex-specific immune signatures may influence treatment efficacy. Future work aimed at defining molecular subtypes of AD based on immune activation patterns may therefore facilitate more precise and personalized therapeutic approaches.

## Conclusions

Together, our findings identify interferon signaling as a sex-biased driver of Alzheimer’s disease pathobiology that preferentially affects females. By integrating human transcriptomic analyses with experimental perturbation in mouse models of AD, we show that amplification of an interferon-responsive state exacerbates neuroinflammatory and neurodegenerative features of the disease, whereas pharmacological suppression of this pathway mitigates neuropathological alterations and preserves cognitive function in female APP/PS1 mice. Transcriptomic profiling across tissue, cell-type, and single-cell resolutions further indicates that microglia represent a major cellular compartment engaging interferon responses in AD. Notably, interferon-associated tissue alterations occur largely independently of changes in amyloid plaque burden, suggesting that interferon signaling modulates disease-associated damage beyond plaque deposition. These results highlight interferon signaling as a modifiable neuroimmune pathway contributing to disease vulnerability and progression, providing a mechanistic framework for female-biased susceptibility to AD.

## Methods

### Sex as a biological variable

Both male and female mice were included in all experimental groups unless otherwise stated, and sex was a central consideration in the study design. Experimental cohorts were balanced by sex, and all transcriptomic, histological, and behavioral analyses were stratified accordingly. This allowed for direct comparison of AD–related phenotypes and immune signaling between male and female animals. Similarly, human transcriptomic datasets from the The Mount Sinai Brain Bank (MSBB) study [33] comprised comparable numbers of male and female samples, allowing sex-stratified differential expression analyses. Collectively, these approaches ensured that sex was rigorously incorporated as a biological variable and systematically addressed throughout the study.

### Mice

APP/PS1 [111] and Cx3cr1^CreERT2^ [112] mice are available at public repositories (RRID:MMRRC_034832-JAX and RRID:IMSR_JAX:021160, respectively). Rela^fl/fl^ mice which harbor loxP sites flanking exons 5–8 of the *Rela* gene, have been previously described (MGI:3,775,205) [113]. Cx3cr1^CreERT2^ mice were crossed with Rela^fl/fl^ mice [113] resulting in the line referred as Cx3cr1^CreERT2^-Rela^fl/fl^. These mice were crossed with APP/PS1 mice to generate Cx3cr1^CreERT2^-Rela^fl/fl^-APP/PS1. In the experiments involving tamoxifen-induced knockouts, we used tamoxifen-treated Rela^fl/fl^-APP/PS1-littermates as controls. All transgenic lines were maintained on a C57BL/6 J genetic background.

### BV2 microglial cell line

Mouse microglial BV2 cells were cultured in high-glucose Dulbecco’s modified Eagle’s medium (DMEM) (Thermo Fisher Scientific, 41965047) supplemented with 10% heat-inactivated fetal bovine serum (FBS) (Thermo Fisher Scientific, 10,270,106) and 1% penicillin–streptomycin (Sigma-Aldrich, P4333). Cells were maintained at 37 °C in a humidified atmosphere with 5% CO_2_ and plated at a density of 3 × 10^5^ cells per well in 6-well plates for stimulation assays. BV2 cells were pre-treated for 5 h with C-176 (7.5 μg/mL; MedChemExpress, HY-112906) or vehicle (corn oil/ethanol, 90:10 v/v), followed by 24 h stimulation with Polyinosinic:polycytidylic acid high molecular weight (poly(I:C), 10 μg/mL; Sigma-Aldrich, P1530-25MG) or endotoxin-free water in the continued presence of C-176 or vehicle. For BV2 experiments, cells from a single culture flask were evenly distributed into multiple wells and randomly assigned to the different experimental conditions. Each condition comprised four independent wells, which were treated and processed independently for gene expression analyses.

### In vivo animal treatments

According to each experiment, animals were treated following ethical guides as described previously. To induce Cre recombination in Cx3cr1^CreERT2^ mice, tamoxifen (TMX) (Sigma-Aldrich, T5648) was dissolved at 25 mg/mL in corn oil (Sigma-Aldrich, C8267) by heating to 50 °C for 1 h under light-protected conditions. Three-month-old mice received two intraperitoneal injections of tamoxifen (75 mg/kg) on alternate days. All downstream experiments were conducted three months after tamoxifen administration to allow turnover of peripheral CX3CR1⁺ cells while maintaining labelling of long-lived microglia [114]. Mice were 6 months old at the time of analysis. Polyinosinic:polycytidylic acid high molecular weight (poly(I:C)) (Sigma-Aldrich, P1530-25MG) was prepared in sterile, endotoxin-free water and administered intraperitoneally at a dose of 12 mg/kg. Mice were sacrificed 24 h post-injection for transcriptomic analyses, and at 24- or 72-h post-injection for histological analyses. For histological analyses, control animals received saline injections and were collected at matching time points (24 h or 72 h post-injection). Saline-treated animals from both time points were pooled for subsequent analyses. The STING inhibitor C-176 (MedChemExpress, HY-112906) was prepared at a concentration of 1.5 mg/mL in a corn oil:ethanol (90:10, v/v). Mice received intraperitoneal (i.p.) injections of C-176 at 10 mg/kg or vehicle on alternate days from 3 to 6 months of age, during the period of APP/PS1 pathology development [36, 37]. For behavioral experiments, the same cohort of animals was used for baseline WT vs AD comparisons and for treatment studies. Vehicle-treated animals served both as baseline controls and as controls for treatment analyses.

### Immunohistochemistry and image analysis

Mice were deeply anesthetized and transcardially perfused with PBS, followed by 4% paraformaldehyde (PFA) (PFA, w/v, dissolved in 0.1 M phosphate buffer, pH 7.4). Brains were post-fixed overnight and cryoprotected in 30% (w/v) sucrose for 48h. Coronal Sects. (50 µm thickness) were obtained using a cryotome and stored in antifreeze solution (30% ethylene glycol, 30% glycerol, 30% distilled water, 10% 10 × phosphate-buffered saline (PBS)) at − 20 °C until processing. Briefly, free-floating sections were washed three times in PBS (5 min each at room temperature (RT)), followed by three washes in PBS-T (0.3% Triton X-100; Sigma-Aldrich, T8787)), and then blocked for 2 h at RT in PBS-T containing 5% newborn calf serum (NCS; Sigma-Aldrich, N4762). Sections were subsequently incubated overnight at 4 °C with primary antibodies diluted in PBS-T with 5% NCS. The following primary antibodies were used: IFITM3 (Proteintech, 11,714–1-AP, 1:500), 6E10 (anti-β-Amyloid, 1–16) (BioLegend, 803,001, 1:1000), c-FOS (Synaptic Systems, 226,308, 1:1000), phosphorylated neurofilament (pNF) (Biolegend, SMI 31P, 1:500), Parvalbumin (Synaptic Systems, 195,004 1:1000), GFAP (Sigma-Aldrich, G3893, 1:500), IBA1 (Wako, 019–19741, 1:500), CD68 (Abcam, ab53444, 1:500), and OC (Sigma-Aldrich, AB2286, 1:5000). On the following day, sections were rinsed six times in PBS-T and incubated for 2 h at RT with fluorophore-conjugated secondary antibodies diluted in PBS-T with 5% NCS, followed by three additional washes in PBS-T and three washes in PBS. The following secondary antibodies were used: Donkey anti-rat IgG H + L Alexa Fluor 488 (Abcam, ab150153), Donkey anti-rabbit IgG H + L Alexa Fluor 647 (Abcam, ab150067), Donkey anti-mouse IgG H + L Alexa Fluor Plus 594 (Thermo Fisher Scientific, A32744) and Donkey anti-guinea pig IgG H + L Alexa Fluor 647 (Abcam, 706–605-148), all at 1:500 dilution. Finally, tissue sections were counterstained with DAPI (Thermo Fisher Scientific, A32733) and mounted onto glass slides using Fluoromount- G (Sigma-Aldrich, T5941). For thioflavin T staining, after secondary antibody staining, sections were incubated with 0.01% Thioflavin T (Sigma-Aldrich, T3516) in water for 5 min, followed by 50% ethanol and PBS washes. For the immunodetection of perineuronal nets (PNNs), after secondary antibody staining, slices were incubated overnight at 4 °C with a solution containing biotinylated *Wisteria floribunda* lectin (WFA) (Vector Laboratories, B-1355–2, 1:300). On the following day, sections were rinsed 3 times in PBS (5 min each) at RT, incubated with a solution of red fluorescent streptavidin (Alexa Fluor™ 555 conjugate) (Thermo Fisher Scientifics, S32356, 1:300) and 5% NCS in PBS for 1 h at RT, and rinsed again 3 times in PBS. Images were acquired using a Zeiss Axio Scan.Z1 slide scanner (20 × objective), and maximum intensity projections were generated from z-stacks. High-resolution images were acquired using a Zeiss LSM 880-Airyscan Elyra PS1 super-resolution inverted confocal microscope. Regions of interest (ROIs) were manually drawn around the dentate gyrus, CA1 region, cortex, or the entire hippocampus. Image quantification was performed using Arivis Vision4D software with standardized macros for thresholding and segmentation. Quantitative analyses of IBA1 and CD68 immunolabelling were performed using Fiji (ImageJ v1.54p, java 8) [115]. Quantification metrics included IBA1^+^ and CD68^+^ area, as well as mean CD68^+^ particle area and density within IBA^+^-segmented regions in CA1 stratum radiatum; total number of cFOS⁺ nuclei in the dentate gyrus; total number of WFA⁺ PNN structures and the percentage of PV^+^ interneurons surrounded by PNNs in CA1; number and total area of GFAP⁺ astrocytic objects; total pNF⁺ area in the CA1 stratum radiatum; total number and area of IFITM3⁺ cells in cortex and hippocampus; and amyloid plaque density, number, and average size of Thioflavin T⁺, 6E10⁺, and OC⁺ amyloid deposits in cortex and hippocampus. For all brain immunohistochemistry analyses, two coronal sections per animal were used, and all quantified values correspond to the average of both sections. Sections were selected systematically based on anatomical criteria, ensuring clear inclusion of the hippocampus in all cases while avoiding excessively rostral or caudal levels, in order to maintain appropriate anatomical correspondence between animals.

### FACS sorting of microglia

Brains were collected and placed on ice in sterile phosphate-buffered saline (PBS). After removal of the meninges, cortical hemispheres were mechanically dissociated in Dounce buffer (15 mM HEPES, 0.5% glucose, HBSS 1x) using a tissue homogenizer (Fisher Scientific, 10,198,611). Myelin was removed by centrifugation through a 25% isotonic Percoll gradient at 800 × g for 15 min at 4°C. The resulting cell pellet was resuspended in PBS, centrifuged, and processed for flow cytometry to isolate microglia as previously described [116]. Briefly, prior to antibody staining, samples were blocked with Fc-block CD16/CD32 (Biolegend, 101,320, 1:50) in FACS Buffer sterile filtered (1% FCS, 2mM EDTA, 25mM HEPES in PBS) for 10 min on ice and protected from light to block nonspecific binding. For cell surface staining, cells were resuspended in the appropriate antibody cocktail and incubated for 30 min on ice protected from light. Primary antibodies used were anti-mouse GFP (Aveslab, GFP-1020, 1:500), anti-mouse Cd11b (BioLegend, 101,235, 1:500), anti-mouse CD45 (BioLegend, 103,106, 1:500). Samples were centrifuged and washed with FACS buffer. Viability was assessed by staining with DAPI (1 μg/mL). Cells were sorted using a BD FACS Aria III cell sorter (BD Bioscience). To sort the cells, a 85-micron nozzle with 4-Way purity mode was used. The gating strategy involved selecting the cell population based on forward scatter area (FSC-A) to discriminate cells by size, and side scatter area (SSC-A) to separate them by granularity or internal complexity. Doublets were then excluded by gating FSC-height (FSC-H) and FSC-A to isolate single cells. This strategy enriches for Cx3cr1⁺ myeloid cells, which in the adult mouse brain are predominantly associated with parenchymal microglia. While a minor contribution of other Cx3cr1⁺ myeloid populations, such as perivascular macrophages, cannot be completely excluded, meningeal macrophages were excluded by removal of the meninges prior to tissue processing. All final analysis and data output were performed using FlowJo software (BD). For RNA-seq experiments, microglia were isolated from the whole adult mouse cortex, yielding 200,000–300,000 cells. Sorted cells were collected directly into RLT buffer (Quiagen, 79216) for subsequent RNA extraction.

### Real-time quantitative PCR (RT-qPCR)

To analyze gene expression in acutely FACS-isolated microglia from the adult mouse hippocampus, RNA was extracted using the RNeasy Mini Kit (Qiagen, 74,104) with on-column DNase digestion (Thermo Fisher Scientific, 18,047,019). For BV2 cells, RNA was isolated using TRIzol reagent (Thermo Fisher Scientific, A33251). For all samples, RNA purity, integrity, and concentration were assessed by spectrophotometry (Nanodrop ND-1000 spectrophotometer) and micro-capillary electrophoresis (2100 Bioanalyzer, Agilent). Complementary DNA was synthesized using the RevertAid First Strand cDNA Synthesis Kit (Thermo Fisher Scientific, EP0442) following the manufacturer’s protocol, and quantitative PCR was performed using EvaGreen Master Mix (Cultek, 755,032) on a QuantStudio 3 system (Applied Biosystems). Relative expression levels were analyzed using the ΔΔCt method [117], with *Gapdh* as the housekeeping gene for normalization. Primer sequences used in RT–qPCR assays are listed in Table S7.

### Human transcriptomic samples (MSBB Cohort)

RNA-seq data from the parahippocampal gyrus (Brodmann area 36) and frontal pole (Brodmann area 10) were obtained from the Mount Sinai Brain Bank (MSBB) via the AMP-AD Knowledge Portal (Synapse ID: syn3159438; metadata: syn29855570; count data: syn27068754). We used processed raw count matrices in which genes with fewer than 1 count per million (CPM) had already been filtered out. For our analyses, we further subset the dataset to include only PHG and FP samples of AD cases aged 60 to 89 years at death. Resequenced samples were handled by excluding unique sequencing batches; when biological replicates from the same individual were available, those with higher RNA integrity (RIN and RIN^2^ scores) were preferentially retained. To reduce sex-related confounding effects, samples were additionally filtered based on the expression of sex-linked genes, resulting in the removal of one sample (Fig. S1, A and B). The final dataset included 34 female and 32 male AD cases from PHG tissue, and 44 females and 40 males from FP tissue. For additional analyses, samples were filtered using the same criteria, selecting either control samples aged 60 to 89 years at death (19 females and 23 males for PHG, and 27 females and 23 males for FP). Statistical tests were carried out to assess data distribution prior analysis, we compared age at death, plaque mean, Braak stage, and RIN across groups stratified by sex and disease status (Fig. S1, C-J). After observing significant differences in the distribution of age at death, we included age at death as a covariate in subsequent differential expression analyses.

### Mouse transcriptomic profiling by RNA-seq

For bulk hippocampal profiling (GSE304521), hippocampi from adult mice were rapidly dissected on ice using fine forceps, and total RNA was extracted individually from each animal using the RNeasy Mini Kit (Qiagen, 74104), with on-column DNase digestion (Thermo Fisher Scientific, 18,047,019). Each transcriptomic profile corresponds to an individual animal. RNA quality was assessed by micro-capillary electrophoresis (Agilent TapeStation, Agilent), and only samples with RNA Integrity Number (RIN) ≥ 7 were used for library construction. Libraries were amplified by PCR, purified, and size-selected to enrich for fragments compatible with Illumina sequencing, followed by quality assessment using fluorometry (Qubit fluorometer, Thermo Fisher Scientific) and micro-capillary electrophoresis (Agilent TapeStation, Agilent). Libraries were prepared by Novogene Europe (Cambridge, UK) using a strand-specific protocol with poly-A selection (Novogene NGS Stranded RNA Library Prep Set (PT044)) and sequenced on an Illumina NovaSeq X Plus platform (PE150). Raw paired-end RNA-seq reads in FASTQ format were processed by Novogene Europe (Cambridge, UK) using their standard bioinformatics pipeline. Briefly, reads with adapter sequences, reads containing poly-N (N > 10%), and reads with high proportion of low-quality bases (Qscore of over 50% bases below 5) were removed using fastp [118]. Quality metrics such as Q20, Q30, and GC content were also calculated during this step. All downstream analyses were based on the resulting high-quality clean reads. Clean reads were aligned to the mm39 mouse reference genome using HISAT2 (v2.2.1; [119]). The reference genome index was built using HISAT2, and gene model annotations were used to enable splice-aware alignment with improved accuracy. Library sizes of primary mapped reads were consistently above 50 million fragments. Gene-level quantification was performed using featureCounts (v2.0.6; [120]). The resulting count tables were used for downstream differential expression analysis.

For microglial transcriptomic profiling in poly(I:C)-treated mice (GSE304362), bulk microglia were isolated from the whole adult mouse cortex 24 h after poly(I:C) or vehicle administration (control), as described above. Total RNA and quality control were performed as described above, using RNeasy Mini Kit (Qiagen, 74,104) and micro-capillary electrophoresis (Agilent TapeStation, Agilent) to ensure RIN ≥ 7. RNA-seq libraries were constructed using Novogene NGS RNA Library Prep Set (PT042). Raw paired-end data were processed in-house following a custom analysis pipeline. Briefly, raw paired-end RNA-seq reads in FASTQ format were first subjected to quality control using FastQC (v0.11.9; https://www.bioinformatics.babraham.ac.uk/projects/fastqc/) to assess base quality and sequence content. Adapter sequences and low-quality bases were then removed using Trim Galore (v0.6.7; https://zenodo.org/records/7598955) in paired-end mode. Post-trimming quality was re-evaluated with FastQC to confirm adapter removal and improved read quality. High-quality reads were aligned to the GRCm38 mouse reference genome using HISAT2 (v2.2.1; [119]) with the –dta option to facilitate downstream transcript assembly. Library sizes of primary mapped reads were consistently above 50 million fragments. Alignment output in SAM format was converted and sorted into BAM format using SAMtools (v1.13; [121]), and subsequently indexed. For genome browser visualization, BAM files were converted to TDF format using IGVTools (v2.5.3), aligned to the mm10 genome build, and visualized in IGV [122]. Gene-level quantification was performed using HTSeq-count (v0.13.5) with parameters –stranded = no, –type = exon, and –order = pos. Gene annotations were obtained from Ensembl release 102 (Mus_musculus.GRCm38.102.chr.gtf). The resulting count tables were used for downstream differential expression analysis.

### Differential gene expression analysis

Differential expression analyses were conducted in R version 4.5.0 (R Core Team, 2025) using DESeq2 (v1.48.1; [123]), considering genes with adjusted P value < 0.05 as significantly differentially expressed. Batch correction was applied when needed using the ComBat_seq() function from the sva package (v3.56.0, [124]). To facilitate the detection of batch effects, outliers, and major sources of variance, Principal Component Analyses were performed on variance-stabilized data (VST for datasets with > 30 samples) or regularized log-transformed data (rlog for datasets with < 30 samples) using the plotPCA() function from the DESeq2 package. The top 500 most variable genes were used for dimensionality reduction. The first two principal components were visualized with ggplot2 (v3.5.2, [125]), and sample labels were added using geom_text_repel() from ggrepel (v0.9.6, [126]) to improve readability. Heatmaps were generated on gene expression counts rlog-transformed using pheatmap (v1.0.13, [127]), with hierarchical clustering applied to genes and samples ordered by experimental design. Rows were scaled using z-scores, and styling was harmonized with the overall plot design. Volcano plots were generated using ggplot2, where the –log₁₀ adjusted p-value was plotted against the log₂ fold change. Genes were color-coded by significance (adj. p < 0.05 or < 0.1) and classified as upregulated, downregulated, or not significant. Key interferon-alpha–related genes were labeled using ggrepel. Optional axis cutoffs were applied to reduce the impact of extreme outliers on plot scaling.

### Functional enrichment analysis

Functional enrichment analyses were performed in predefined gene sets, using Enrichr [128] and the MSigDB “Hallmark” 2020 gene set collection [34]. Whereas differential expression analysis in the PHG identified 158 upregulated and 90 downregulated genes in females relative to males (adjusted p < 0.05), the FP yielded a more restricted set of significantly regulated transcripts, comprising 15 upregulated and 20 downregulated genes (Table S1). Given this reduced number of differentially expressed genes, enrichment analyses in the FP were performed using a ranked list of the top 200 transcripts. In functional enrichment plots, bar length represents the Enrichr combined score and bars are colored according to statistical significance. The Enrichr combined score is a ranking metric calculated by multiplying the log-transformed p-value (derived from Fisher’s exact test) by the z-score (deviation from the expected rank), thereby integrating statistical significance with the magnitude of enrichment. Gene Set Enrichment Analysis (GSEA) was performed to identify coordinated shifts in predefined gene sets, using the GSEA_4.4.0 desktop application [35], with the following parameters: 1,000 phenotype permutations, No_Collapse, and a fixed permutation seed (149). Ranked gene lists (.rnk files) were generated from DESeq2 output using log_2_ fold change as the ranking metric and processed with tidyverse tools (v2.0.0, [129]) without additional filtering. Enrichment was tested against the MSigDB “Hallmark” gene sets for both human (v2025.1.Hs) and mouse (v2025.1.Mm) [34]. GSEA plots were recreated in R using ggplot2, cowplot (v1.2.0, [130]), and gridExtra (v2.3, [131]) to maintain stylistic consistency across visual outputs.

### Reanalysis of published snRNA-seq data

Single-nucleus RNA-sequencing data from the hippocampi of 8-month-old male APPPS1 [132] (n = 3) and controls (WT; n = 2) mice were obtained from the Gene Expression Omnibus (GEO; accession GSE173242) [46]. Raw count matrices were imported into R using the Read10X() function and processed with Seurat (v5.3.0). A total of 44,997 nuclei were initially analyzed (cells per library: APPPS1_1 = 6,425; APPPS1_2 = 10,175; APPPS1_3 = 11,573; WT1 = 7,961; WT2 = 8,863). Putative doublets were identified and removed using scDblFinder (v1.21.2). Quality-control filtering excluded nuclei with fewer than 200 or more than 3,000 detected genes, as well as nuclei with mitochondrial transcript content exceeding 5%. After filtering, 40,232 high-quality nuclei were retained for downstream analyses (cells per library: APPPS1_1 = 5,910; APPPS1_2 = 9,083; APPPS1_3 = 10,220; WT1 = 6,915; WT2 = 8,104). Data were normalized and clustered following the standard Seurat workflow, and clustering was performed using a resolution of 0.4. An interferon alpha (IFN-α) gene module score was computed using the AddModuleScore() function in Seurat, based on genes from the MSigDB Hallmark “interferon alpha response” gene set [34].

### Mouse behavioral testing

All behavioral assays were conducted during the light phase under consistent illumination (~ 20 lx) in a dedicated testing suite. Mice were acclimated to the testing room for at least 30 min before each test. Behavior was recorded and analyzed using SMART 3.0 software (Panlab). We used adult mutant and control littermates of both sexes. For the *open field* (OF) test, mice were placed in the center of a 40 × 40 × 40 cm arena and allowed to explore freely for 15 min. Total distance traveled and time spent in the center zone were recorded as measures of locomotor activity and anxiety-like behavior, respectively. The *elevated plus maze* (EPM) consisted of two open and two closed arms (30 × 5 cm), elevated 50 cm above the floor. Mice were placed in the center facing an open arm and allowed to explore for 5 min. The percentage of time spent and number of entries into open arms were calculated. To evaluate spatial working memory spontaneous alternation was measured using the *Y-maze*. Briefly, the transparent symmetrical Y-maze consisted of three arms (30 × 5 × 15 cm). Three objects of similar size (∼20 cm3) were situated surrounding the maze at ∼15–20 cm outside the walls and mice were allowed to freely explore the maze for 8 min. An alternation was defined as sequential entry into all three arms (triad). Percent alternation = (Number of alternations/[Total arm entries − 2]) × 100. To study short-term spatial memory, we used the *T-maze*. The apparatus consisted of three arms, two of them situated at 180° from each other and the third situated at 90° with respect to the other ones representing the stem arm of the T. All three arms were 45 cm long. In the training trial, one arm was closed (novel arm) and mice were placed in the stem arm of the T (home arm) and allowed to explore this arm and the other available arm (familiar arm) for 10 min, after which they were returned to the home cage. After an inter-trial interval of 1 h mice were placed in the stem arm of the T-maze and allowed to freely explore all three arms for 5 min (testing phase). The time in the new arm ∗ 100/total time in the two arms at 180° was the parameter evaluated. In the *novel object location* (NOLT) memory task, mice were habituated to a 40 × 40 cm arena for 15 min. Different distal visual cues were allocated in the walls of the arena. On day 2, two identical objects were presented during a 10-min familiarization phase. After a 24-h delay, one object was relocated to a novel position (test phase), and mice were allowed to explore for 5 min. The discrimination index was calculated as: (Time_novel − Time_familiar)/Total_exploration_time. Associative memory was assessed using *fear conditioning paradigm*. A 25 × 25 × 25 cm black chamber system was used (Panlab, Spain). On Day 1, animals were allowed to habituate for 5 min. After habituation, a tone (90 dB) was presented for 30 s. During the final 2 s of the tone, a 0.4 mA foot shock was administered for 1–2 s and the mouse was allowed to remain 30 additional seconds in the chamber. This procedure was repeated two times, and the mouse remained 2 additional minutes in the chamber before removing it. On Day 2, the contextual memory test was conducted. The mouse was placed in the chamber for 5 min, with no tone presented, and freezing behaviour was recorded. Cued memory was assessed on day 3 in a novel context, consisting of a 5-min baseline followed by a 30-s tone presentation. Freezing was defined as the absence of all movement except respiration for > 1 s. Animals were tracked and recorded with the Packwin V2.0.08 software (Panlab, Spain).

### Statistics

Data are presented as mean ± standard error of the mean (SEM), unless otherwise indicated. Statistical analyses were performed using GraphPad Prism (version 7) or R (version 4.2 or later). For comparisons between two groups, unpaired two-tailed Student’s t tests were used. Comparisons involving more than two groups were analyzed using one-way ANOVA followed by Bonferroni post hoc correction. For non-normally distributed data, two tailed Mann–Whitney U test was applied. For behavioral assessment with two between-subject factors, data were analyzed by two-way ANOVA with interaction terms evaluated. Post hoc multiple-comparisons tests used Tukey’s correction. For NOLT discrimination index, post hoc comparisons were performed using Fisher’s LSD. For NOLT exploration time (old vs new location), data were analyzed using two-way repeated-measures ANOVA (within-subject factor: location), followed by Bonferroni-corrected post hoc tests for within-group comparisons between old versus new locations. Correlations were assessed using Pearson’s correlation coefficient. A p-value less than 0.05 was considered statistically significant. Specific statistical tests and sample sizes (n) for each experiment are reported in the figure legends.

## Supplementary Information

Supplementary Material 1: Fig. S1. Sex-associated differences in interferon signaling in Alzheimer’s disease. (A) Principal component analysis (PCA) of RNA-seq data from parahippocampal gyrus (PHG) samples of patients aged 60-90 years from the MSBB cohort. One sample clustering with the opposite sex group was excluded from downstream analyses. (B) Heatmap showing expression of representative sex chromosome genes across PHG samples from AD patients aged 60-90 years. The excluded sample is indicated in bold. (C-J) Violin plots showing plaque mean (C, D), Braak stage (E, F), age at death (G, H), and RNA integrity number (RIN; I, J) in AD and control samples stratified by brain region (PHG and frontal pole, FP) and sex (PHG AD, n = 34 females and 32 males; PHG control, n = 19 females and 23 males; FP AD, n = 44 females and 40 males; FP control, n = 27 females and 23 males). Two-group comparisons were analyzed using the two-tailed Mann-Whitney U test (C, E, G, I) and sex × condition effects were analyzed using two-way ANOVA followed by Tukey’s HSD post hoc test (D, F, H, J) (*p < 0.05; **p < 0.01; ***p < 0.001; ****p < 0.0001). (K–M) Functional enrichment analysis of significantly differentially expressed genes (adj. p < 0.05) in PHG tissue from AD patients. Bar plots show enriched gene sets in combined AD samples (K), male AD samples (L), and female AD samples (M). (N) Volcano plot of differential gene expression in PHG tissue from control individuals comparing females and males. (O-P) GSEA plot of the MSigDB Hallmark “interferon alpha response” gene set based on differential expression analysis between females and males control samples in PHG (O) and FP (P).

Supplementary Material 2: Fig. S2. High-resolution representative images of immunofluorescence labelling used throughout the study. (A) Amyloid-β plaques labelled with the 6E10 antibody. (B) Fibrillar amyloid deposits detected with Thioflavin T (ThT). (C) Conformational amyloid species labelled with the OC antibody. (D) IBA1 and CD68 immunolabelling. (E) GFAP immunolabelling. (F) cFOS immunolabelling. (G) Perineuronal nets (PNN) labelled with Wisteria floribunda agglutinin (WFA) in parvalbumin-positive (PV) interneurons. (H) Phosphorylated neurofilament (pNF) immunolabelling. Nuclei are counterstained with DAPI in all panels. Images were acquired using a super-resolution inverted confocal microscope (Zeiss LSM 880 Airyscan Elyra PS.1) with a 63× objective. Scale bars: 15 µm.

Supplementary Material 3: Fig. S3. Neuropathological and transcriptomic characterization of male and female APP/PS1 mice. (A-C) Average amyloid plaque size in cortex (CTX) and hippocampus (HPC) measured using 6E10 immunostaining (A), Thioflavin T staining (ThT; B), and OC immunostaining (C) (6E10: n = 5 males and 7 females; ThT: n = 6 males and 6 females; OC: n = 5 males and 6 females). (D) Quantification of CD68^+^ area and mean CD68^+^ particle area in the CA1 stratum radiatum (n = 6 per group). (E) Quantification of GFAP^+^ area in the cortex (n = 5 males and 6 females). (F) Quantification of WFA^+^ cells in CA1 (n = 6 per group). (G) Volcano plot showing differential gene expression in the hippocampus of APP/PS1 (AD) versus wild-type (Ctrl) mice. Significantly upregulated genes in APP/PS1 mice are shown in green, downregulated genes in dark grey, and non-significant genes in light grey (adj. p < 0.05; n = 16 per group). Top differentially expressed genes are labelled. (H) Functional enrichment analysis of genes upregulated in the hippocampus of 6-month-old APP/PS1 mice (adj. p < 0.05). (I) GSEA plot of the MSigDB Hallmark “interferon alpha response” gene set based on differential expression analysis in the hippocampus of female versus male APP/PS1 mice (n = 8 per group). (J) Feature plot showing normalized *P2ry12 *expression. (K) Feature plot showing interferon alpha response gene set module scores. Graphs in (A-F) represent data distribution as dots and bars indicating mean ± SEM. Statistical significance was assessed using two-tailed Mann-Whitney U test (ns, not significant; *p < 0.05; **p< 0.01).

Supplementary Material 4: Fig. S4. Poly(I:C)-induced interferon signaling and histopathological alterations in wild-type mice. (A) Principal component analysis (PCA) of hippocampal RNA sequencing (RNA-seq) data from 3-month-old wild-type C57BL/6 mice 24 h after poly(I:C) (12 mg/kg, i.p.) or saline treatment (control). Points are colored by treatment and shaped by sex (poly(I:C), n = 4 males and 5 females; control, n = 5 males and 4 females). (B) Quantification of CD68^+^ area and mean CD68^+^ particle area in the CA1 stratum radiatum (n = 6 per group). (C) Quantification of GFAP immunolabelling in the cortex (CTX) after saline or poly(I:C) treatment at 24 h and 72 h (saline, n = 5; poly(I:C) 24 h, n = 7; poly(I:C) 72 h, n = 7). (D) Quantification of WFA^+^ cells in CA1 after saline or poly(I:C) treatment at 24 h and 72 h (saline, n = 6; poly(I:C) 24 h, n = 7; poly(I:C) 72 h, n = 7). (E) Representative gating strategy for acutely isolated microglia from adult Cx3cr1::CreERT2-EYFP. (F) PCA of microglial transcriptomes from control and poly(I:C)-treated mice (poly(I:C), n = 3; control, n = 4). (G) GSEA plot of the MSigDB Hallmark “interferon alpha response” gene set based on differential expression analysis in adult microglia following poly(I:C) treatment. Graphs in (B-F) represent data distribution as dots and bars indicating mean ± SEM. Statistical significance was assessed using one-way ANOVA followed by Bonferroni’s multiple-comparisons test. ns, not significant (*p < 0.05; **p< 0.01; ***p< 0.001; ****p< 0.0001).

Supplementary Material 5: Fig. S5. Neuropathological and transcriptomic characterization of genetically enhanced interferon signaling in APP/PS1 mice. (A) Principal component analysis (PCA) of hippocampal RNA sequencing (RNA-seq) data from 6-month-old APP/PS1 (AD) and microglia-specific *Rela* knockout (AD hiIFN) mice, (n = 4 males and 4 females per group). (B) GSEA plot of the MSigDB Hallmark “interferon alpha response” gene set based on differential expression analysis in the hippocampus of AD hiIFN versus AD mice. (C) Heatmap displaying normalized expression of interferon-alpha-related genes in hippocampal tissue from male and female AD and AD hiIFN mice. (D) Quantification of CD68^+^ area and mean CD68^+^ particle area in the CA1 stratum radiatum (n = 6 per group). (E) Quantification of GFAP^+^ area in the cortex (n = 6 per group). (F) Quantification of WFA^+^ cells in CA1 (n = 6 per group). (G) Average amyloid plaque size in cortex (CTX) and hippocampus (HPC) measured using 6E10 immunostaining (AD, n = 6; AD hiIFN, n = 5). (H, I) Representative images and quantification of amyloid-β (Aβ) plaque burden with thioflavin T staining (ThT; H) and OC immunostaining (I) in cortex (CTX) and hippocampus (HPC) of AD and AD hiIFN mice at 6 months of age (ThT: AD, n = 8; AD hiIFN = 6; OC: AD, n = 6; AD hiIFN, n = 5). Nuclei were counterstained with DAPI. Scale bars: 500 μm; inset 100 μm. (J, K) Average amyloid plaque size in cortex (CTX) and hippocampus (HPC) measured using Thioflavin T staining (ThT; J) and OC immunostaining (K) (ThT: AD, n = 8; AD hiIFN = 5; OC: AD, n = 6; AD hiIFN = 5). Graphs represent data distribution as dots and bars indicating mean ± SEM. Statistical significance was assessed using two-tailed Mann-Whitney U test (ns, not significant; *p < 0.05; **p < 0.01).

Supplementary Material 6: Fig. S6. STING inhibition in APP/PS1 female mice. (A) qPCR analysis of selected interferon-related genes (*Irf7*, *Ifitm3*, and *Stat2*) and *Il10* in BV2 microglial cells treated with the STING inhibitor C-176 (7.5 μg/mL) and/or poly(I:C) (10 μg/mL) (n = 4 per condition). Statistical significance was assessed using two-way ANOVA followed by Bonferroni-corrected post hoc tests. (B) Heatmap displaying normalized expression of interferon-alpha-related genes in hippocampal tissue from male and female AD and AD loIFN mice (AD M, n = 8; AD F, n = 8; AD loIFN M, n = 4; AD loIFN F, n = 4; M = male, F = female). (C) Quantification of CD68^+^ area and mean CD68^+^ particle area in the CA1 stratum radiatum (n = 6 per group). (D) Quantification of GFAP^+^ area in the cortex (n = 6-7 per group). (E) Quantification of WFA^+^ cells in CA1 (n = 6-7 per group). (F) Average amyloid plaque size in cortex (CTX) and hippocampus (HPC) of AD and AD loIFN female mice measured using 6E10 immunostaining (AD, n = 6; AD loIFN, n = 7). (G, H) Representative images and quantification of amyloid-β (Aβ) plaque burden using thioflavin T staining (ThT; G) and OC immunostaining (H) in cortex (CTX) and hippocampus (HPC) of AD and AD loIFN female mice at 6 months of age (ThT: AD, n= 6-7; AD loIFN = 7-8; OC: AD, n = 6; AD loIFN = 7). Nuclei were counterstained with DAPI. Scale bars: 500 μm; inset 100 μm. (I, J) Average amyloid plaque size in cortex (CTX) and hippocampus (HPC) measured using Thioflavin T staining (ThT; I) and OC immunostaining (J) (ThT: AD, n = 6-7; AD loIFN = 7-8; OC: AD, n = 6; AD loIFN = 7). Graphs represent data distribution as dots and bars indicating mean ± SEM. Statistical significance for panels (C-J) was assessed two-tailed Mann-Whitney U test (C-J) (ns, not significant; **p < 0.01;***p < 0.001; ****p < 0.0001).

Supplementary Material 7: Fig. S7. STING inhibition in APP/PS1 male mice. (A) Volcano plot of differential gene expression analysis in the hippocampus of AD loIFN versus AD male mice (AD M, n = 8; AD loIFN M, n = 4). (B) Functional enrichment analysis using the top 100 downregulated genes ranked by adjusted p-value from differential expression analysis comparing AD loIFN versus AD male mice. (C-G) Quantification of immunostaining in the hippocampus of AD and AD loIFN male mice (n = 6-9 per group): IBA^+^ area and CD68^+^ metrics, including particle density, total area, and mean particle area, in the CA1 stratum radiatum (C); GFAP in the hippocampus (D); cFOS in the dentate gyrus (E); PV and WFA labelling of perineuronal nets (PNNs) in the CA1 region (F); pNF in the stratum radiatum (G). (H-M) Amyloid plaque density (H-J) and size (K-M) in cortex (CTX) and hippocampus (HPC) of AD and AD loIFN male mice measured using 6E10 immunostaining (H, K), thioflavin T staining (ThT; I, L) and OC immunostaining (J, M) (n = 7-9 per group). Graphs represent data distribution as dots and bars indicating mean ± SEM. Statistical significance was assessed using the two-tailed Mann-Whitney U test (ns, not significant; *p < 0.05; **p < 0.01).

Supplementary Material 8: Fig. S8. Behavioral assessment following STING inhibition in male and female APP/PS1 mice. (A, F, I) Exploration time during the test phase of the novel object location test (NOLT). (B, K, O) Distance traveled in the center of the arena in the open field test. (C, J, L, P) Discrimination index in T-maze or NOLT tasks, as indicated. (D, M, Q) Contextual fear memory and (E, N, R) cued fear memory during the fear conditioning test. (G) Time spent in open arms in the elevated plus maze (EPM). (H) Alternation rate in the Y-maze. Group abbreviations: male (M), female (F), wild-type (WT), APP/PS1 (AD), vehicle (Veh), and C-176 (C176). Graphs represent data distribution as dots and bars indicating mean ± SEM. Statistical analyses were performed using two-way ANOVA followed by Tukey’s HSD post hoc test (B, D, E, G, H, K, M-O, Q, R). Discrimination index analyses (C, J, L, P) used Fisher’s LSD post hoc comparisons. Exploration time in the NOLT (A, F, I; old vs new location) was analyzed using two-way repeated-measures ANOVA (within-subject factor: location), followed by Bonferroni-corrected post hoc tests for within-group comparisons. ns, not significant; *p< 0.05; **p< 0.01; ***p< 0.001; ****p< 0.0001.

Supplementary Material 9: Table S1. Sex-associated differential gene expression in the parahippocampal gyrus (PHG) and frontal pole (FP) of Alzheimer’s disease patients and controls.

Supplementary Material 10: Table S2. Whole-hippocampus transcriptomic profiling in APP/PS1 mice: disease and sex effects.

Supplementary Material 11: Table S3. Differential gene expression in the whole hippocampus 24 h after systemic poly(I:C) (i.p.) administration in adult mice.

Supplementary Material 12: Table S4. Differential gene expression in acutely isolated cortical microglia 24 h after systemic poly(I:C) (i.p.) administration in adult mice.

Supplementary Material 13: Table S5. Differential gene expression in APP/PS1 mice with adult-induced microglia-specific*Rela* conditional knockout.

Supplementary Material 14: Table S6. Differential gene expression in the whole hippocampus of male and female APP/PS1 mice following chronic C-176 administration.

Supplementary Material 15: Table S7. Oligonucleotide sequences used for RT-qPCR.

## Data Availability

The transcriptomics data sets generated in this study can be accessed at the GEO public repository using the accession number GSE304521 and GSE304362. In addition, publicly available single-cell RNA-seq data (GSE173242) were also reanalyzed as part of this study [46]. Human RNA-seq data used in this study were obtained from the publicly available The Mount Sinai Brain Bank (MSBB) study, hosted on the AD Knowledge Portal (https://adknowledgeportal.org/). The dataset is accessible via Synapse (https://www.synapse.org/): syn3159438; metadata: syn29855570; count data: syn27068754). The results published here are in whole or in part based on data obtained from the AD Knowledge Portal (https://adknowledgeportal.org/). These data were generated from postmortem brain tissue collected through the Mount Sinai VA Medical Center Brain Bank and were provided by Dr. Eric Schadt from Mount Sinai School of Medicine. Processed data and code used for statistical analysis, gene expression processing, and Fig. generation are available upon request from the corresponding author.

## Abbreviations

AD: Alzheimer’s disease
Aβ: Amyloid-β
CTX: Cortex
EPM: Elevated plus maze
FP: Frontal pole
GWAS: Genome-wide association study
HPC: Hippocampus
MSBB: Mount Sinai Brain Bank
NCS: Newborn calf serum
NOLT: Novel object location test
PBS: Phosphate-buffered saline
PFA: Paraformaldehyde
PHG: Parahippocampal gyrus
pNF: Phosphorylated neurofilament
PNNs: Perineuronal nets
poly(I:C): Polyinosinic:polycytidylic acid
ROIs: Regions of interest
RT: Room temperature
TMX: Tamoxifen
WFA: Wisteria floribunda lectin
