# Supplementary figures and images for: Sex-dependent interferon signaling contributes to female-biased vulnerability in Alzheimer’s disease
Source: J Neuroinflammation. 2026 May 4;23:217. doi: 10.1186/s12974-026-03840-0 (PMC13312654; doi:10.1186/s12974-026-03840-0)

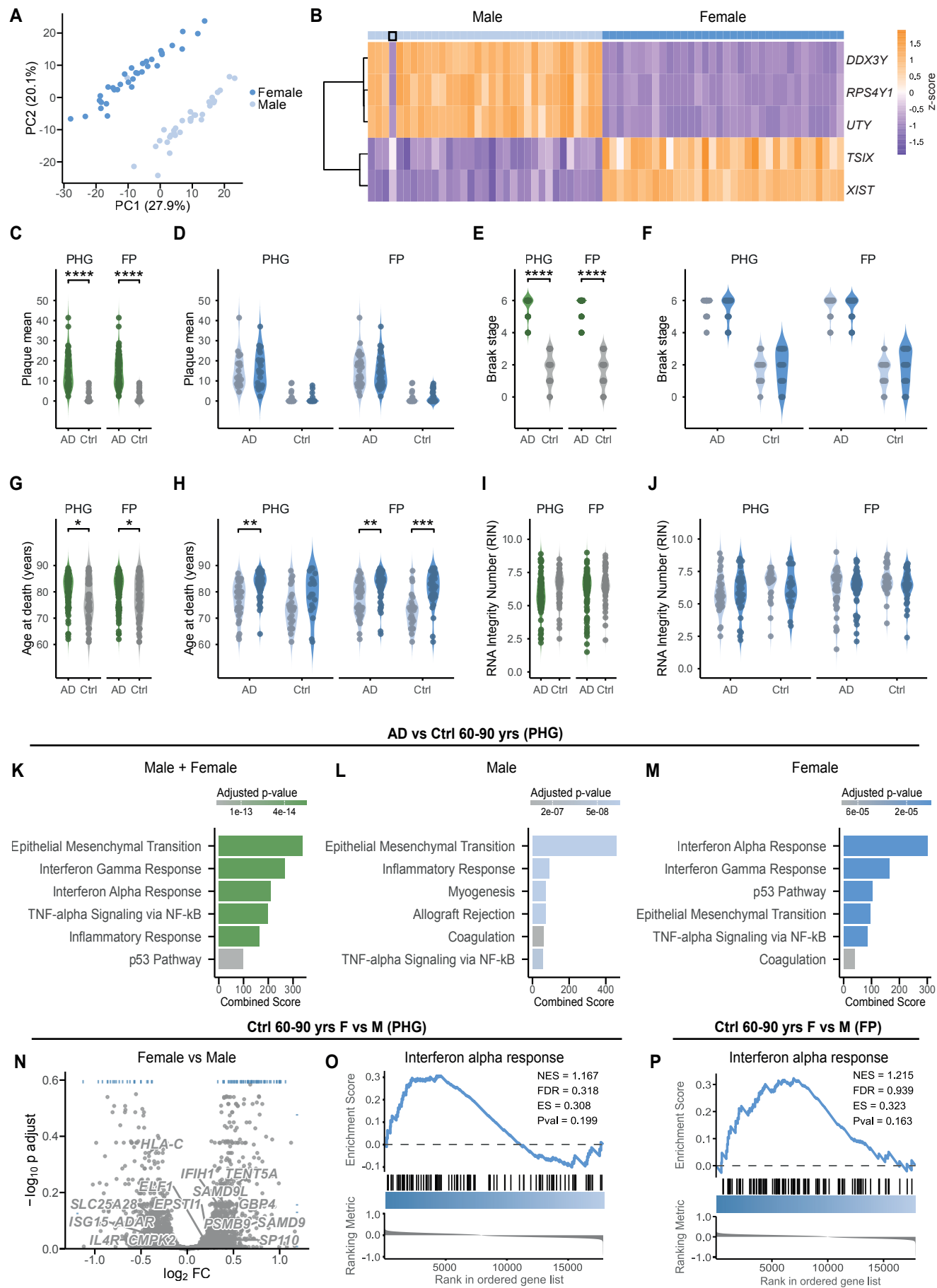

**Fig. S1**

Supplement: Supplementary file 1 — Supplementary Material 1: Fig. S1. Sex-associated differences in interferon signaling in Alzheimer’s disease. (A) Principal component analysis (PCA) of RNA-seq data from parahippocampal gyrus (PHG) samples of patients aged 60-90 years from the MSBB cohort. One sample clustering with the opposite sex group was excluded from downstream analyses. (B) Heatmap showing expression of representative sex chromosome genes across PHG samples from AD patients aged 60-90 years. The excluded sample is indicated in bold. (C-J) Violin plots showing plaque mean (C, D), Braak stage (E, F), age at death (G, H), and RNA integrity number (RIN; I, J) in AD and control samples stratified by brain region (PHG and frontal pole, FP) and sex (PHG AD, n = 34 females and 32 males; PHG control, n = 19 females and 23 males; FP AD, n = 44 females and 40 males; FP control, n = 27 females and 23 males). Two-group comparisons were analyzed using the two-tailed Mann-Whitney U test (C, E, G, I) and sex × condition effects were analyzed using two-way ANOVA followed by Tukey’s HSD post hoc test (D, F, H, J) (*p < 0.05; **p < 0.01; ***p < 0.001; ****p < 0.0001). (K–M) Functional enrichment analysis of significantly differentially expressed genes (adj. p < 0.05) in PHG tissue from AD patients. Bar plots show enriched gene sets in combined AD samples (K), male AD samples (L), and female AD samples (M). (N) Volcano plot of differential gene expression in PHG tissue from control individuals comparing females and males. (O-P) GSEA plot of the MSigDB Hallmark “interferon alpha response” gene set based on differential expression analysis between females and males control samples in PHG (O) and FP (P). [file 12974_2026_3840_MOESM1_ESM.pdf]

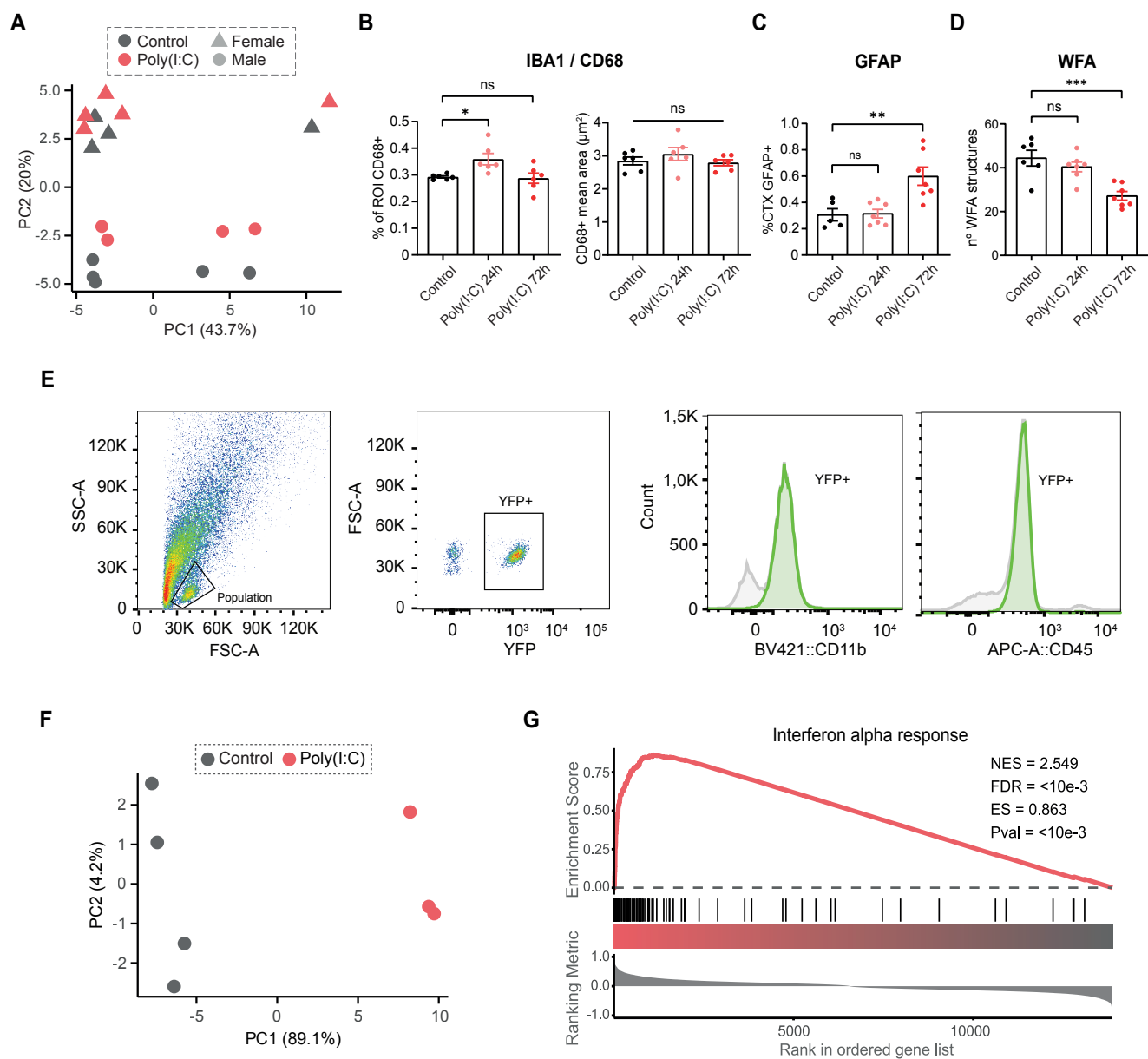

**Fig. S4**

Supplement: Supplementary file 4 — Supplementary Material 4: Fig. S4. Poly(I:C)-induced interferon signaling and histopathological alterations in wild-type mice. (A) Principal component analysis (PCA) of hippocampal RNA sequencing (RNA-seq) data from 3-month-old wild-type C57BL/6 mice 24 h after poly(I:C) (12 mg/kg, i.p.) or saline treatment (control). Points are colored by treatment and shaped by sex (poly(I:C), n = 4 males and 5 females; control, n = 5 males and 4 females). (B) Quantification of CD68+ area and mean CD68+ particle area in the CA1 stratum radiatum (n = 6 per group). (C) Quantification of GFAP immunolabelling in the cortex (CTX) after saline or poly(I:C) treatment at 24 h and 72 h (saline, n = 5; poly(I:C) 24 h, n = 7; poly(I:C) 72 h, n = 7). (D) Quantification of WFA+ cells in CA1 after saline or poly(I:C) treatment at 24 h and 72 h (saline, n = 6; poly(I:C) 24 h, n = 7; poly(I:C) 72 h, n = 7). (E) Representative gating strategy for acutely isolated microglia from adult Cx3cr1::CreERT2-EYFP. (F) PCA of microglial transcriptomes from control and poly(I:C)-treated mice (poly(I:C), n = 3; control, n = 4). (G) GSEA plot of the MSigDB Hallmark “interferon alpha response” gene set based on differential expression analysis in adult microglia following poly(I:C) treatment. Graphs in (B-F) represent data distribution as dots and bars indicating mean ± SEM. Statistical significance was assessed using one-way ANOVA followed by Bonferroni’s multiple-comparisons test. ns, not significant (*p < 0.05; **p< 0.01; ***p< 0.001; ****p< 0.0001). [file 12974_2026_3840_MOESM4_ESM.pdf]

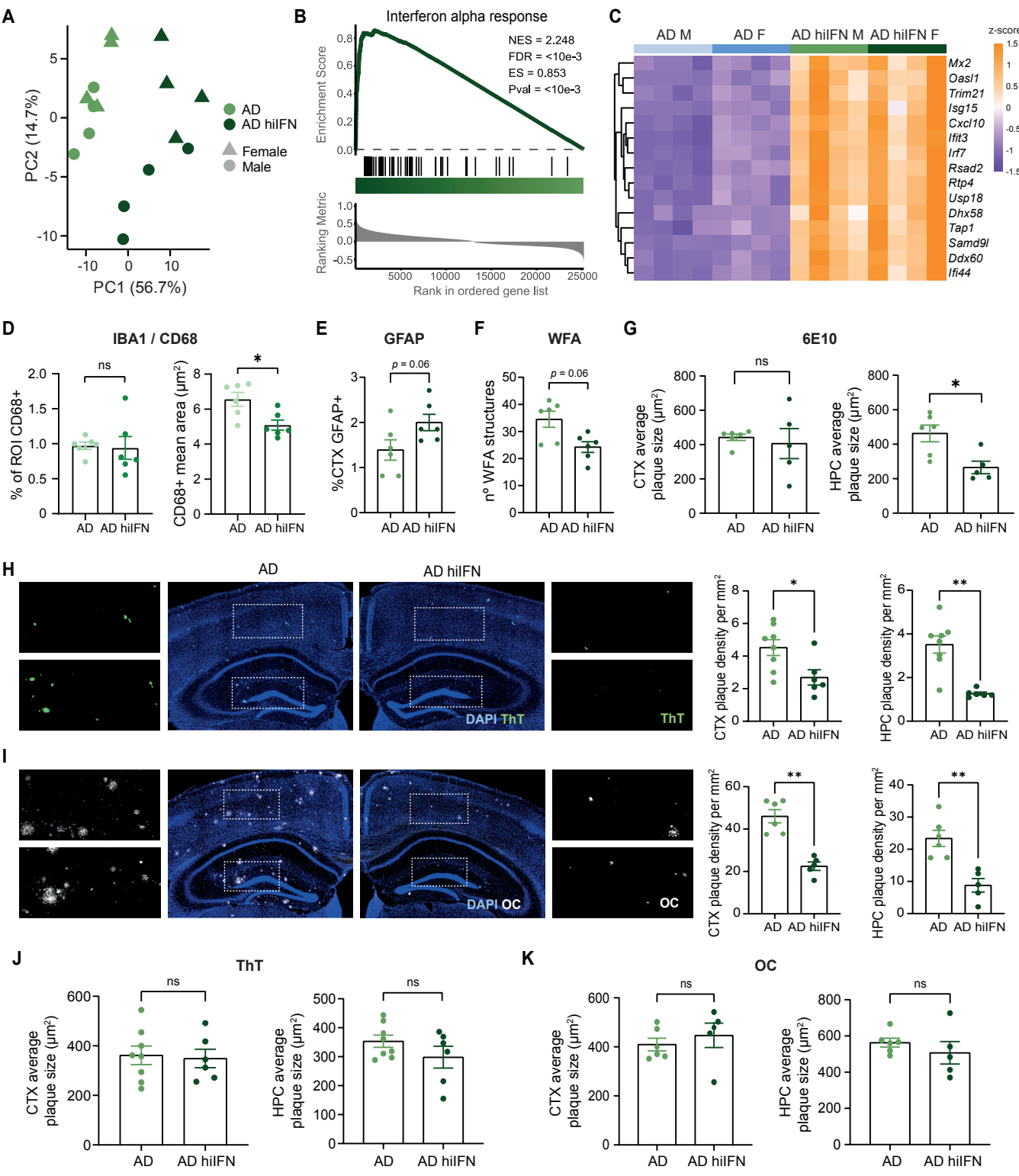

Fig. S5

Supplement: Supplementary file 5 — Supplementary Material 5: Fig. S5. Neuropathological and transcriptomic characterization of genetically enhanced interferon signaling in APP/PS1 mice. (A) Principal component analysis (PCA) of hippocampal RNA sequencing (RNA-seq) data from 6-month-old APP/PS1 (AD) and microglia-specific Rela knockout (AD hiIFN) mice, (n = 4 males and 4 females per group). (B) GSEA plot of the MSigDB Hallmark “interferon alpha response” gene set based on differential expression analysis in the hippocampus of AD hiIFN versus AD mice. (C) Heatmap displaying normalized expression of interferon-alpha-related genes in hippocampal tissue from male and female AD and AD hiIFN mice. (D) Quantification of CD68+ area and mean CD68+ particle area in the CA1 stratum radiatum (n = 6 per group). (E) Quantification of GFAP+ area in the cortex (n = 6 per group). (F) Quantification of WFA+ cells in CA1 (n = 6 per group). (G) Average amyloid plaque size in cortex (CTX) and hippocampus (HPC) measured using 6E10 immunostaining (AD, n = 6; AD hiIFN, n = 5). (H, I) Representative images and quantification of amyloid-β (Aβ) plaque burden with thioflavin T staining (ThT; H) and OC immunostaining (I) in cortex (CTX) and hippocampus (HPC) of AD and AD hiIFN mice at 6 months of age (ThT: AD, n = 8; AD hiIFN = 6; OC: AD, n = 6; AD hiIFN, n = 5). Nuclei were counterstained with DAPI. Scale bars: 500 μm; inset 100 μm. (J, K) Average amyloid plaque size in cortex (CTX) and hippocampus (HPC) measured using Thioflavin T staining (ThT; J) and OC immunostaining (K) (ThT: AD, n = 8; AD hiIFN = 5; OC: AD, n = 6; AD hiIFN = 5). Graphs represent data distribution as dots and bars indicating mean ± SEM. Statistical significance was assessed using two-tailed Mann-Whitney U test (ns, not significant; *p < 0.05; **p < 0.01). [file 12974_2026_3840_MOESM5_ESM.pdf]

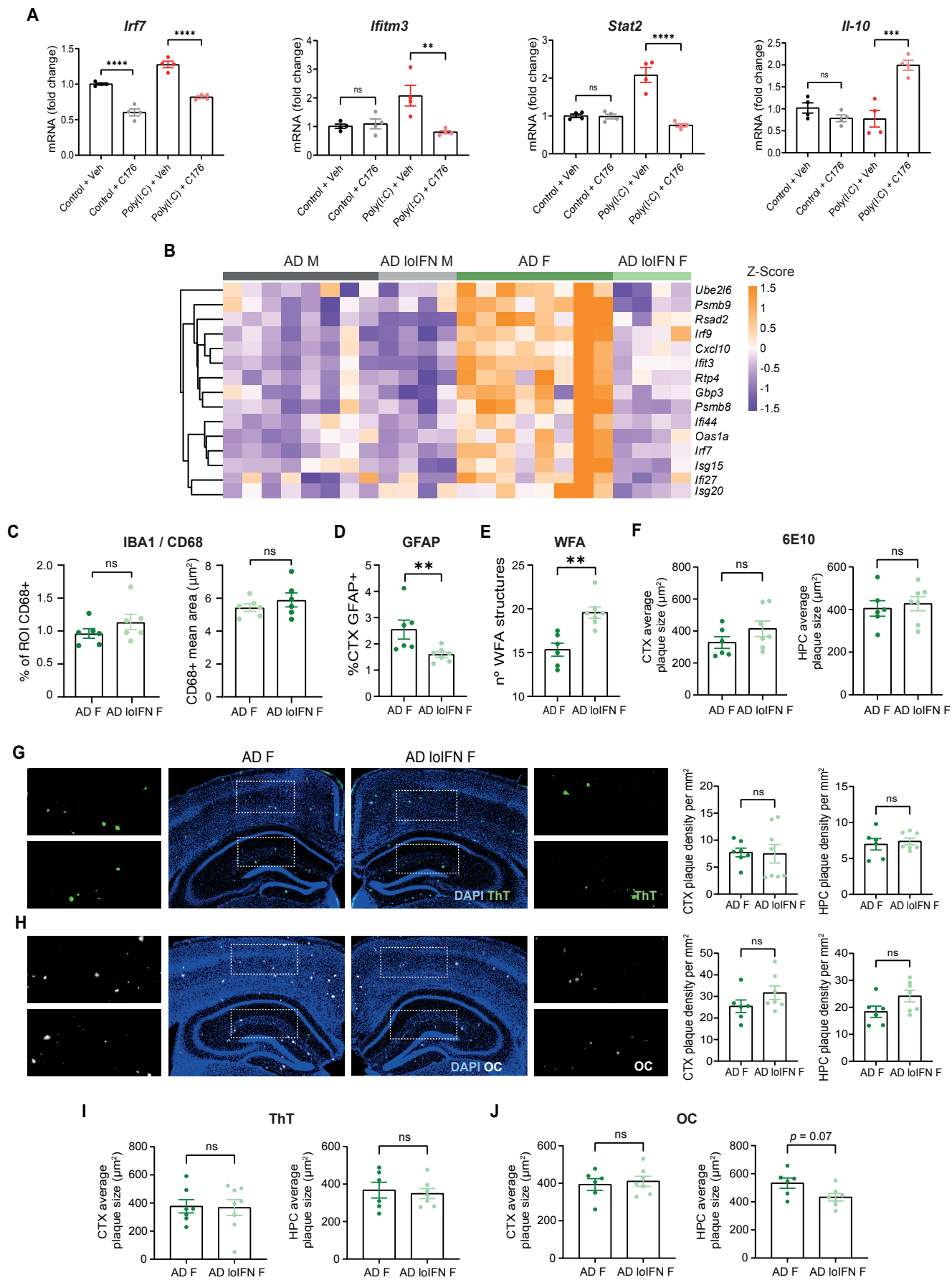

**Fig. S6**

Supplement: Supplementary file 6 — Supplementary Material 6: Fig. S6. STING inhibition in APP/PS1 female mice. (A) qPCR analysis of selected interferon-related genes (Irf7, Ifitm3, and Stat2) and Il10 in BV2 microglial cells treated with the STING inhibitor C-176 (7.5 μg/mL) and/or poly(I:C) (10 μg/mL) (n = 4 per condition). Statistical significance was assessed using two-way ANOVA followed by Bonferroni-corrected post hoc tests. (B) Heatmap displaying normalized expression of interferon-alpha-related genes in hippocampal tissue from male and female AD and AD loIFN mice (AD M, n = 8; AD F, n = 8; AD loIFN M, n = 4; AD loIFN F, n = 4; M = male, F = female). (C) Quantification of CD68+ area and mean CD68+ particle area in the CA1 stratum radiatum (n = 6 per group). (D) Quantification of GFAP+ area in the cortex (n = 6-7 per group). (E) Quantification of WFA+ cells in CA1 (n = 6-7 per group). (F) Average amyloid plaque size in cortex (CTX) and hippocampus (HPC) of AD and AD loIFN female mice measured using 6E10 immunostaining (AD, n = 6; AD loIFN, n = 7). (G, H) Representative images and quantification of amyloid-β (Aβ) plaque burden using thioflavin T staining (ThT; G) and OC immunostaining (H) in cortex (CTX) and hippocampus (HPC) of AD and AD loIFN female mice at 6 months of age (ThT: AD, n= 6-7; AD loIFN = 7-8; OC: AD, n = 6; AD loIFN = 7). Nuclei were counterstained with DAPI. Scale bars: 500 μm; inset 100 μm. (I, J) Average amyloid plaque size in cortex (CTX) and hippocampus (HPC) measured using Thioflavin T staining (ThT; I) and OC immunostaining (J) (ThT: AD, n = 6-7; AD loIFN = 7-8; OC: AD, n = 6; AD loIFN = 7). Graphs represent data distribution as dots and bars indicating mean ± SEM. Statistical significance for panels (C-J) was assessed two-tailed Mann-Whitney U test (C-J) (ns, not significant; **p < 0.01;***p < 0.001; ****p < 0.0001). [file 12974_2026_3840_MOESM6_ESM.pdf]

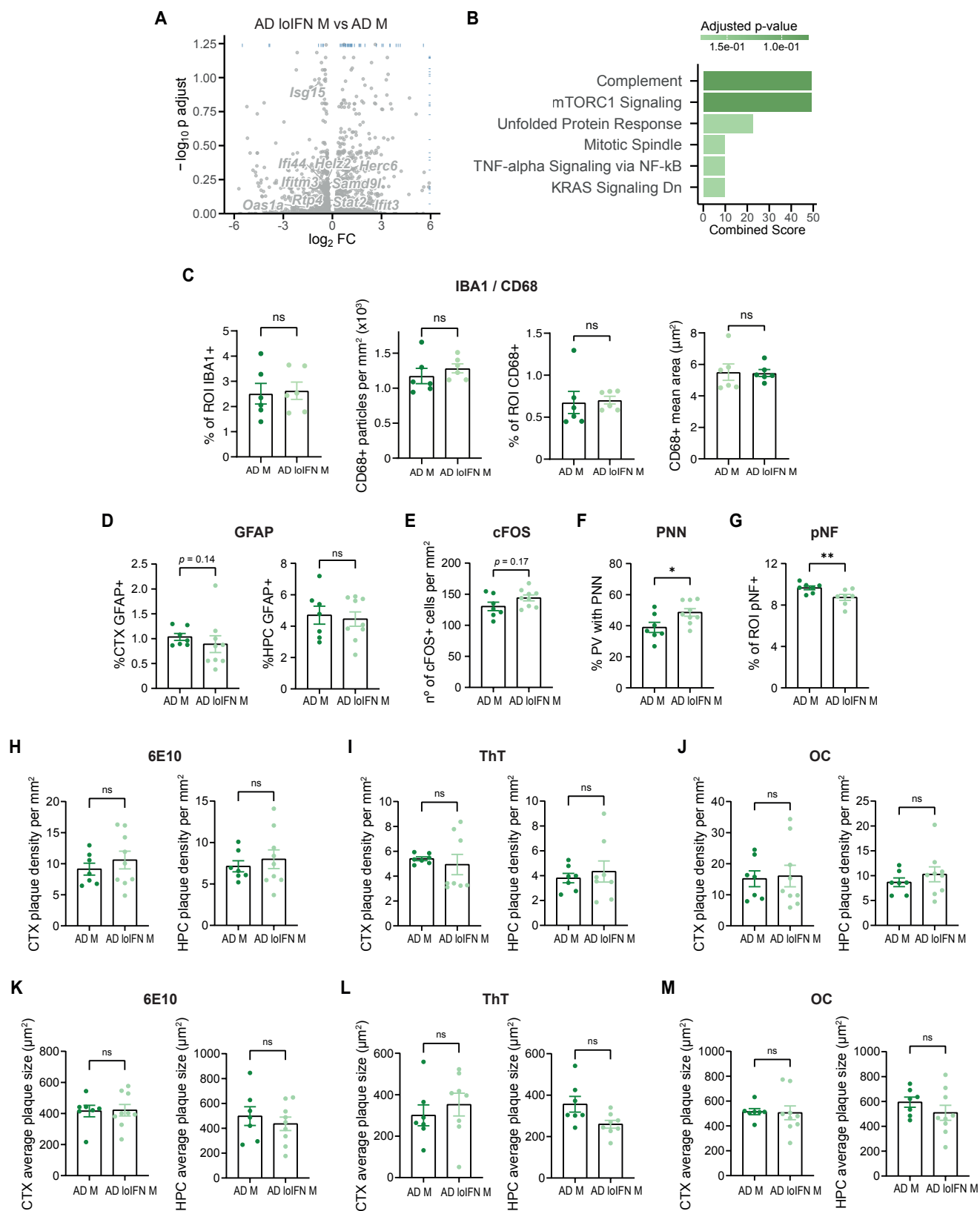

**Fig. S7**

Supplement: Supplementary file 7 — Supplementary Material 7: Fig. S7. STING inhibition in APP/PS1 male mice. (A) Volcano plot of differential gene expression analysis in the hippocampus of AD loIFN versus AD male mice (AD M, n = 8; AD loIFN M, n = 4). (B) Functional enrichment analysis using the top 100 downregulated genes ranked by adjusted p-value from differential expression analysis comparing AD loIFN versus AD male mice. (C-G) Quantification of immunostaining in the hippocampus of AD and AD loIFN male mice (n = 6-9 per group): IBA+ area and CD68+ metrics, including particle density, total area, and mean particle area, in the CA1 stratum radiatum (C); GFAP in the hippocampus (D); cFOS in the dentate gyrus (E); PV and WFA labelling of perineuronal nets (PNNs) in the CA1 region (F); pNF in the stratum radiatum (G). (H-M) Amyloid plaque density (H-J) and size (K-M) in cortex (CTX) and hippocampus (HPC) of AD and AD loIFN male mice measured using 6E10 immunostaining (H, K), thioflavin T staining (ThT; I, L) and OC immunostaining (J, M) (n = 7-9 per group). Graphs represent data distribution as dots and bars indicating mean ± SEM. Statistical significance was assessed using the two-tailed Mann-Whitney U test (ns, not significant; *p < 0.05; **p < 0.01). [file 12974_2026_3840_MOESM7_ESM.pdf]

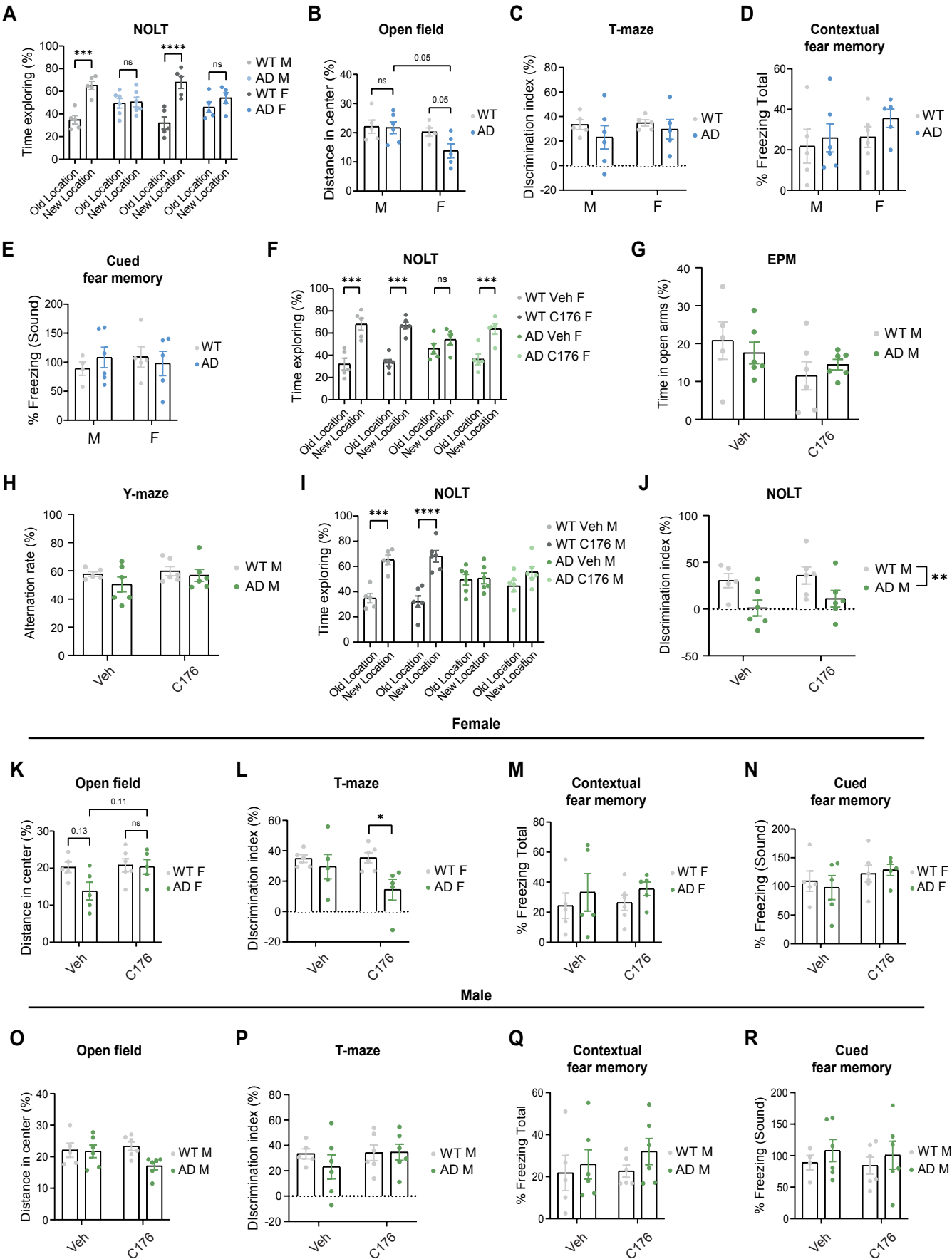

Fig. S8

Supplement: Supplementary file 8 — Supplementary Material 8: Fig. S8. Behavioral assessment following STING inhibition in male and female APP/PS1 mice. (A, F, I) Exploration time during the test phase of the novel object location test (NOLT). (B, K, O) Distance traveled in the center of the arena in the open field test. (C, J, L, P) Discrimination index in T-maze or NOLT tasks, as indicated. (D, M, Q) Contextual fear memory and (E, N, R) cued fear memory during the fear conditioning test. (G) Time spent in open arms in the elevated plus maze (EPM). (H) Alternation rate in the Y-maze. Group abbreviations: male (M), female (F), wild-type (WT), APP/PS1 (AD), vehicle (Veh), and C-176 (C176). Graphs represent data distribution as dots and bars indicating mean ± SEM. Statistical analyses were performed using two-way ANOVA followed by Tukey’s HSD post hoc test (B, D, E, G, H, K, M-O, Q, R). Discrimination index analyses (C, J, L, P) used Fisher’s LSD post hoc comparisons. Exploration time in the NOLT (A, F, I; old vs new location) was analyzed using two-way repeated-measures ANOVA (within-subject factor: location), followed by Bonferroni-corrected post hoc tests for within-group comparisons. ns, not significant; *p< 0.05; **p< 0.01; ***p< 0.001; ****p< 0.0001. [file 12974_2026_3840_MOESM8_ESM.pdf]
